# Synergistic Effects of Mesoporous Structure and Oxygen Vacancies in SnO_2_ for Enhanced CO_2_ Electroreduction

**DOI:** 10.1002/smsc.70268

**Published:** 2026-03-30

**Authors:** Yuguo Zhao, Shoushuang Huang, Yong Yan, Robert Boyd, Zesheng Liu, Mats Fahlman, Mikhail Vagin, Magnus Odén, Emma M. Björk

**Affiliations:** ^1^ Nanostructured Materials Department of Physics, Chemistry and Biology (IFM) Linköping University Linköping Sweden; ^2^ Division of Molecular Surface Physics & Nanoscience Department of Physics, Chemistry and Biology (IFM) Linköping University Linköping Sweden; ^3^ State Key Laboratory of Materials Low‐Carbon Recycling Center of Excellence for Environmental Safety and Biological Effects Department of Chemistry College of Chemistry and Life Science Beijing University of Technology Beijing P. R. China; ^4^ Laboratory of Organic Electronics Department of Science and Technology Linköping University Norrköping Sweden; ^5^ Wallenberg Initiative Materials Science for Sustainability Department of Science and Technology Linköping University Norrköping Sweden

**Keywords:** *OCHO adsorption, CO_2_ electroreduction, mesoporous structure, water dissociation

## Abstract

Electrocatalytic CO_2_ reduction (CO_2_RR) into value‐added chemicals represents a promising strategy for sustainable CO_2_ utilization. This strategy relies on nanoscale structural engineering to gain desired CO_2_RR catalyst performance, which is insufficiently understood. For example, how the pore structure, defect distribution, and surface reconstruction can be used to promote catalytic activity and material stability is not clarified. Here, we investigate how mesopores and oxygen vacancies (V_O_) synergistically regulate the CO_2_RR behavior of SnO_2_. Mesoporous SnO_2_ (M‐SnO_2_) synthesized hydrothermally shows enhanced mesoporosity and a higher specific surface area (59 vs. 21 m^2^ g^−1^) than bulk SnO_2_ (B‐SnO_2_), achieving a Faradaic efficiency (FE) of 50.9% for formate at –1.15 V vs. reversible hydrogen electrode (RHE) and improved durability (FE loss: 13.0% vs. 55.8% after 12 h). Electrochemical analysis, in situ spectroscopy, and density functional theory (DFT) calculations reveal that mesostructure facilitates CO_2_ adsorption, charge transfer, stabilizes *OCHO intermediates, and lowers the reaction energy barrier via V_O_ in M‐SnO_2_. In addition, it is shown that mesostructure promotes formation of V_O_, which stabilizes the oxidation state of Sn and contributes to improved stability of the catalyst. These findings establish the synergistic roles of mesoporous structure and V_O_ for optimizing Sn‐based CO_2_RR catalysts and offer guidance for rational design of efficient CO_2_RR electrocatalysts.

## Graphical abstract

Mesopores in SnO_2_ increase the number of active sites and oxygen vacancies, strengthen intermediate stabilization, and improve structural stability, enhancing CO_2_ to formate conversion.

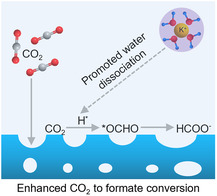

## 1 Introduction

Electrochemical CO_2_ reduction reaction (CO_2_RR) presents an attractive route for converting CO_2_ (including biogenic CO_2_ sources) into value‐added chemicals using renewable electricity, addressing critical challenges in energy storage and climate change mitigation [1, 2, 3]. CO_2_RR can yield a variety of products, ranging from C_1_ species, such as CO and formate, to C_2+_ compounds, such as ethylene and ethanol [4]. While C_2+_ products often are considered more valuable to industry, formation of these compounds requires complex multi‐electron transfer steps and significant energy input for C—C bond formation, limiting the production efficiency [5]. Formate, on the other hand, presents a more favorable thermodynamic formation profile and is widely used in a wide range of industries, such as pharmaceutics, metallurgy, and leather tanning [6]. Moreover, recent studies suggest that producing formate or CO as key intermediates is more economically viable than targeting C_2+_ products, owing to their higher selectivity, production rates, and feasibility for large‐scale implementation [7].

Post‐transition‐metal‐based electrocatalysts, including tin (Sn), indium (In), bismuth (Bi), gallium (Ga), and lead (Pb), have demonstrated promising selectivity toward formate production [8]. Among them, Sn‐based catalysts have generated particular interest due to their low cost, abundance, and environmental compatibility. However, conventional SnO_2_ catalysts typically suffer from limited CO_2_RR activity and poor stability, necessitating strategies to optimize their electronic and structural properties. Several approaches have been explored, including heterometallic doping, such as Cu‐SnO_2_ [9], SnIn‐3 [10], Sn_0.80_Bi_0.20_@Bi‐SnO_*x*_ [11], and CeO_2_–SnO_2_ [12], which enhances charge transfer and water dissociation during CO_2_RR. Additionally, defect engineering, particularly the introduction of oxygen vacancies (V_O_), has been shown to facilitate CO_2_ activation, stabilize key reaction intermediates, and tune product selectivity [13]. There are several strategies to introduce and tailor the V_O_ in the catalysts. These include metal doping, which can introduce charge imbalance and lattice distortion, calcination under reducing atmosphere, which facilitates oxygen removal from the lattice, and the construction of porous structure [9, 14, 15]. In the latter case, when pores are present, the surface area will increase, leading to additional surface atoms with low coordination numbers. As a result, the binding energy of surface oxygen decreases, making it easier to remove it. In addition, when using surfactants in the synthesis, their decomposition during calcination may result in a locally reducing atmosphere that contributes to the formation of V_O_ [16].

In addition to compositional modifications, structural engineering of SnO_2_ has emerged as a key factor in optimizing catalyst performance. Tailoring crystal facets and introducing mesoporosity (pores with sizes of 2 – 50 nm ) can enhance the exposure of active sites and modulate the adsorption behavior of key reaction intermediates [4, 14]. While mesoporous structures are commonly associated with improved mass transport, growing evidence indicates that their influence extends beyond diffusion enhancement, also significantly affecting product selectivity [15, 17, 18]. Various strategies have been developed to construct mesoporous metal oxides, including soft‐templating [19, 20], hard‐templating [21, 22], and anodization[23, 24]. Among them, soft‐templating methods have been extensively explored due to their versatility in controlling pore size, morphology, and framework composition through the cooperative assembly between inorganic precursors and structure‐directing agents [20]. Mesoporous materials have either intra or interparticle pores. In the case of interparticle porosity materials, the material is composed of numerous small particles connected to form a network or chain. Studies suggest that the impact of pore structure cannot be solely attributed to mass transfer effects, but that porosity‐related features, such as surface defects, variations in metals oxidation states, and alterations in local geometric and electronic environments are likely to play pivotal roles in tailoring catalytic activity and selectivity [15, 25]. Nevertheless, most current studies remain phenomenological, lacking comprehensive mechanistic understanding from both experimental and theoretical perspectives. This knowledge gap hinders the rational design of porous SnO_2_‐based catalysts with optimized structure–performance relationships. Thus, understanding how the presence of mesopores stabilizes Sn oxidation states and influences the binding of CO_2_RR intermediates is essential for advancing the development of efficient catalysts.

Herein, we investigate the synergistic effects of mesostructure and V_O_ on the CO_2_RR to formate performance of SnO_2_ catalysts by comparing bulk SnO_2_ (B‐SnO_2_) with mesoporous SnO_2_ (M‐SnO_2_). The results demonstrate that M‐SnO_2_ exhibits higher FE and better stability compared to B‐ SnO_2_. In situ characterization reveals that the porous structure of M‐SnO_2_ enhances the adsorption of CO_2_, water dissociation, and *OCHO, a key intermediate in formate production. Furthermore, mesopore walls contain V_O_ that stabilizes the oxidation state of Sn, thereby improving the long‐term stability of the catalyst. Density functional theory (DFT) calculations further investigate the influence of V_O_, showing that V_O_ in M‐SnO_2_ lowers the activation energy barrier for CO_2_RR to formate. This study demonstrates that synergistic effects between mesostructure and V_O_ modulate the catalytic behavior of SnO_2_, offering valuable insights into the rational design of efficient metal oxide‐based electrocatalysts for CO_2_ reduction.

## 2 Results and Discussion

### 2.1 Structural Characterization of SnO_2_ Catalysts

**FIGURE 1:**
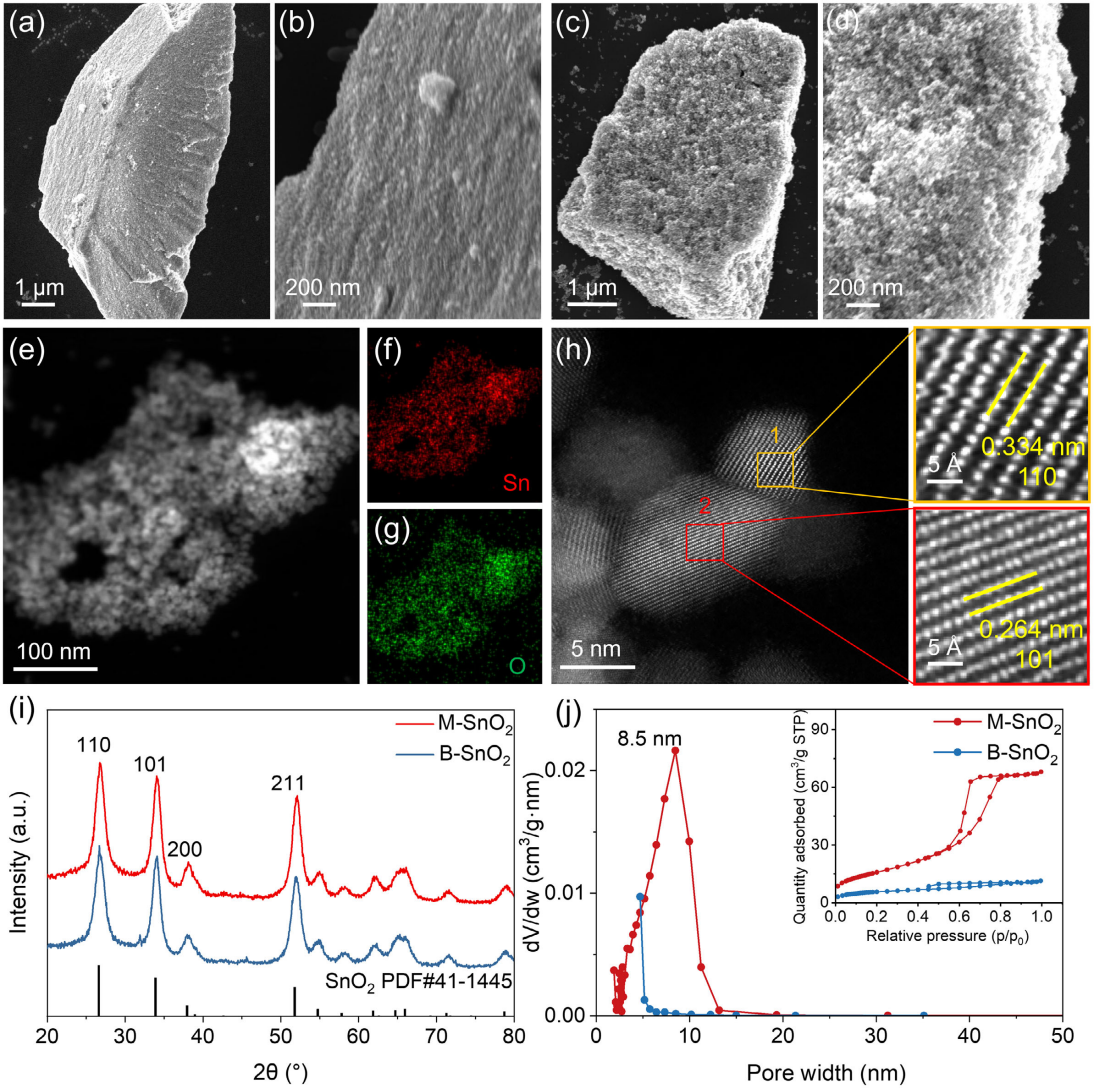
Structural characterization of B‐SnO_2_ and M‐SnO_2_. (a,b) SEM micrographs of B‐SnO_2_. (c,d) SEM micrographs of M‐SnO_2_. (e) HAADF–STEM micrograph of M‐SnO_2_. (f,g) Corresponding STEM‐EDS elemental mapping. (h) High‐resolution HAADF–STEM micrograph of M‐SnO_2_, with two enlarged regions highlighting structural details. (i) X‐ray diffractograms, indicating the crystal structure. (j) Pore size distribution with N_2_ physisorption isotherms shown in the inset.

The morphology of the two SnO_2_ samples is presented in the scanning electron microscopy (SEM) micrographs (Figure 1a–d). B‐SnO_2_ consists of dense nonporous particles, whereas M‐SnO_2_ exhibits a mesoporous structure formed by the assembly of very fine particles. HAADF–STEM imaging and the corresponding elemental mapping (Figure 1e–g), along with energy‐dispersive X‐ray spectroscopy (EDS) analysis (Table S1), further confirm the presence of a porous framework in M‐SnO_2_ with interparticle pores likely forming a three‐dimensional mesoporous network in each nanoparticle assembly, and a homogeneous distribution of Sn and O throughout the sample. The high‐resolution (HR)‐HAADF–STEM micrograph of M‐SnO_2_ (Figure 1h) shows that all particles have a high crystalline order, illustrated by SnO_2_ 110 and 101 lattice planes in the magnified regions. Additional HAADF–STEM micrographs from different regions reveal a porous morphology arising from the aggregation of nanoscale SnO_2_ particles, which is consistent with the SEM observations. Fast Fourier transform (FFT) patterns further validate the presence of the 110 and 101 fringes (Figure S1). Notably, distinct grain boundaries are observed between adjacent crystalline domains in M‐SnO_2_, indicating its mesostructured nature while being highly crystalline.

X‐ray diffraction (XRD) confirms that both samples exhibit diffraction peaks characteristic of tetragonal rutile‐phase SnO_2_ (PDF #41‐1445), with prominent 110, 101, and 211 reflections and no detectable secondary phases (Figure 1i). Despite their identical crystalline phase, the two materials display markedly different textural properties, as revealed by N_2_ physisorption measurements (Figure 1j). M‐SnO_2_ exhibits a type‐IV isotherm with a well‐defined H_2_ hysteresis loop, indicative of a mesoporous structure, whereas B‐SnO_2_ also shows a type‐IV curve with an H_4_ hysteresis loop, characteristic of slit‐like pores between particles [26, 27]. The Brunauer–Emmett–Teller (BET) specific surface area of M‐SnO_2_ (59 m^2^ g^−1^) is higher than that of B‐SnO_2_ (21 m^2^ g^−1^). This enhancement mainly originates from the mesoporous structure of M‐SnO_2_, while the surface area of B‐SnO_2_ is likely due to the presence of micropores and disordered slit‐like voids. Figure 1j further illustrates that M‐SnO_2_ features a uniform mesoporous structure with a pore size of 8.5 nm, whereas B‐SnO_2_ has no distinct pore size distribution.

**FIGURE 2:**
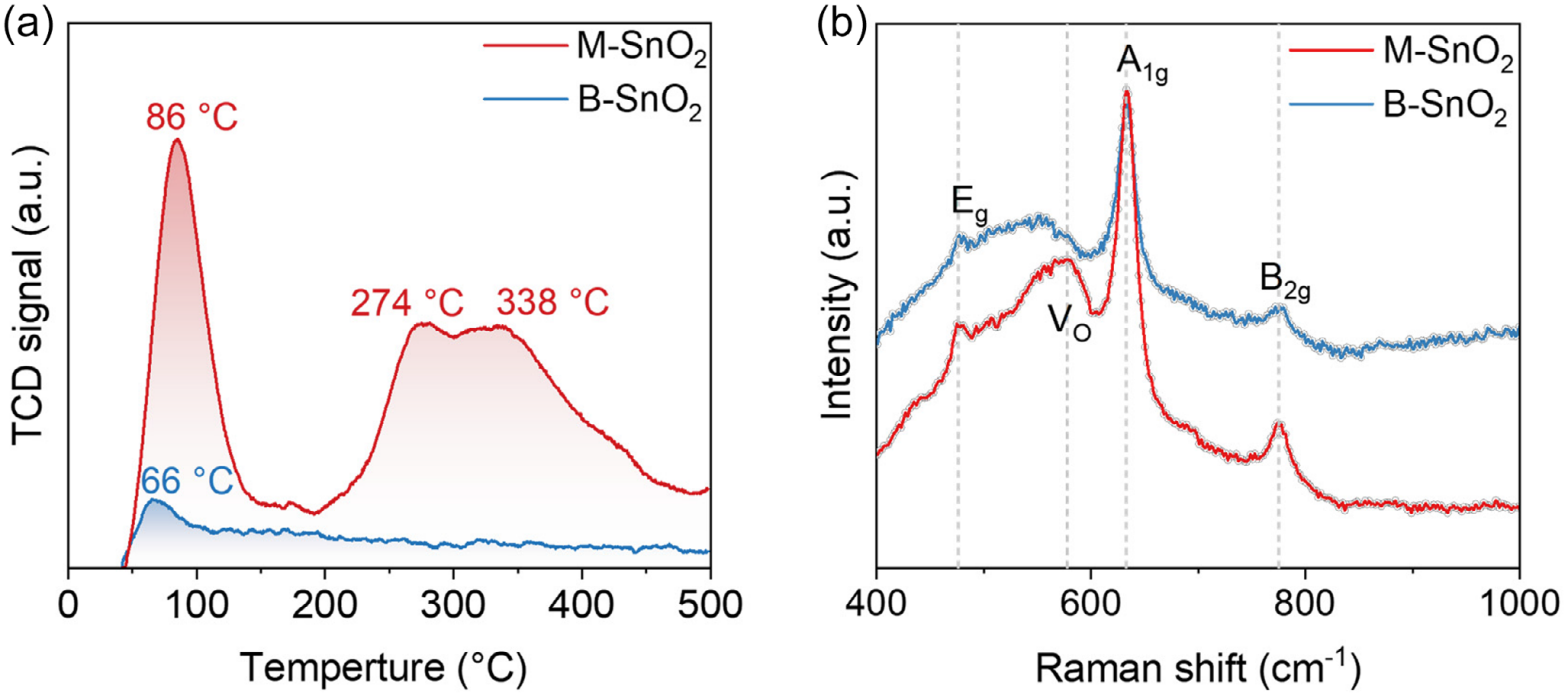
Textural and surface properties of M‐SnO_2_ and B‐SnO_2_. (a) CO_2_‐TPD profiles, illustrating differences in CO_2_ adsorption behavior. (b) Raman spectra highlighting vibrational modes and oxygen vacancy‐related features.

The CO_2_ adsorption capacity of the materials was further evaluated by CO_2_ temperature‐programmed desorption measurements (CO_2_‐TPD, Figure 2a). M‐SnO_2_ exhibits a distinct desorption peak at ∼86°C, attributed to CO_2_ physisorption, together with broader features at higher temperatures (274°C–338°C) associated with chemisorbed CO_2_ species [28]. The substantially larger CO_2_ desorption peak area of M‐SnO_2_ compared to B‐SnO_2_ indicates an enhanced CO_2_ adsorption capacity. Normalization by the specific surface area (Figure S2) suggests that this enhancement cannot be solely attributed to surface area differences, highlighting the role of the mesoporous structure in improving CO_2_ accessibility [26].

To gain insight into the mechanism responsible for this behavior, Raman spectroscopy was employed to probe lattice vibrations and defect‐related features (Figure 2b). Both M‐SnO_2_ and B‐SnO_2_ exhibit characteristic Raman modes of rutile SnO_2_, including the E_g_, A_1g_, and B_2g_ modes. A feature at ∼578 cm^−1^ in M‐SnO_2_, commonly associated with oxygen vacancy‐related defects, is observed [29, 30]. Combined UV–vis, Tauc, and electron paramagnetic resonance (EPR) analyses consistently indicate that M‐SnO_2_ possesses a higher concentration of surface oxygen vacancy‐related defects than B‐SnO_2_. A detailed analysis of defect‐related spectroscopic signatures is provided in Figure S3 and Supplementary Note S1. Overall, these clearly demonstrate the enhanced CO_2_ adsorption capability of M‐SnO_2_ and fundamental variations in the electron structure, which can affect CO_2_RR performance.

### 2.2 CO_2_RR Performance and Reaction Kinetics in Mesopores

**FIGURE 3:**
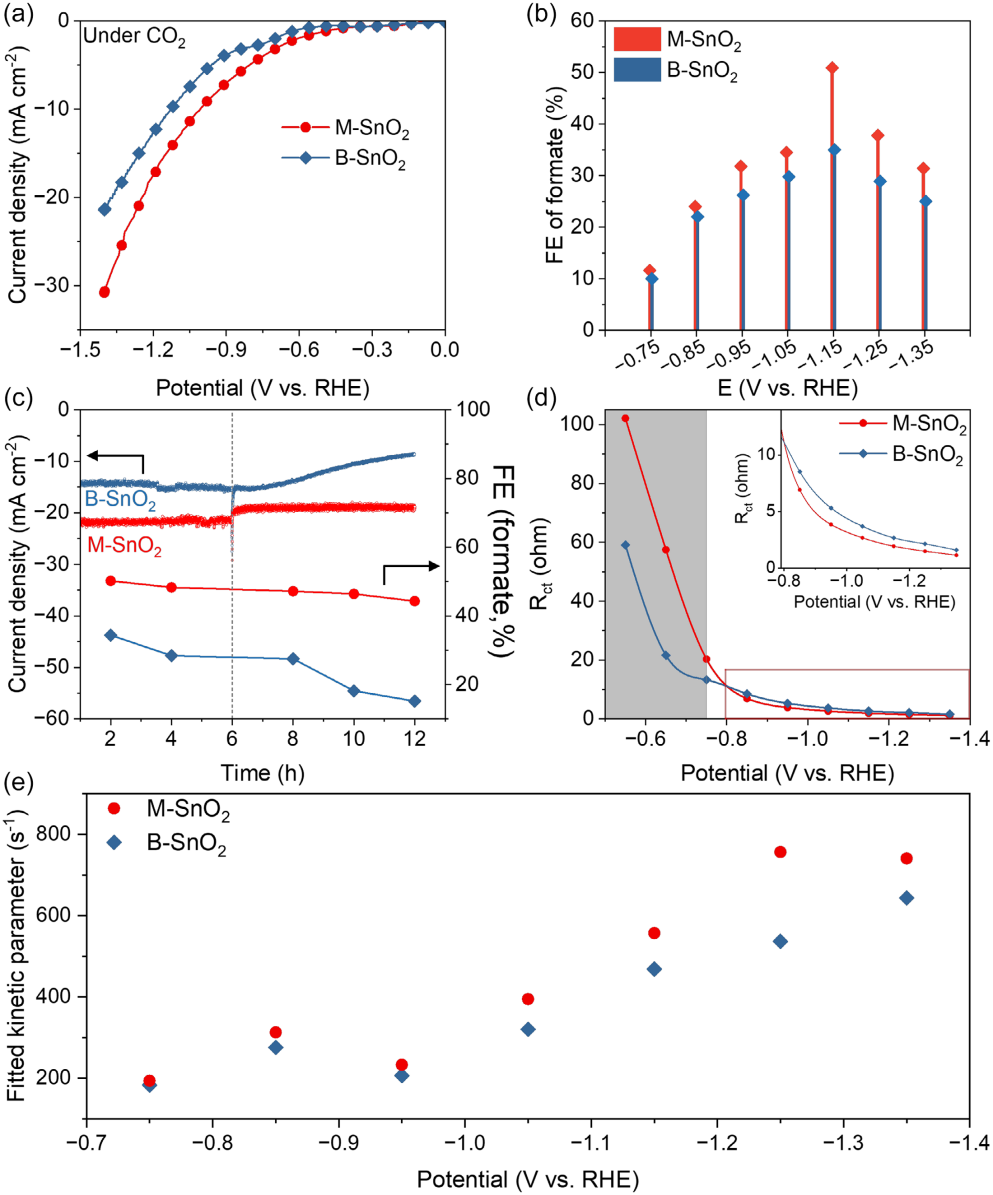
Electrochemical CO_2_RR performance and reaction kinetics of M‐SnO_2_ and B‐SnO_2_. (a) LSV curves in CO_2_‐saturated 0.5 M KHCO_3_. (b) FEs of formate at different applied potentials. (c) Long‐term stability test over 12 h, with the vertical dashed line indicating electrolyte refreshment. (d) Dependence of charge transfer resistance (R_ct_) obtained from R‐CPE circuit. (e) Dependence of the kinetic parameter on different applied potential, obtained as the reciprocal of the RC constant (Figure S13b).

The electrocatalytic performance of SnO_2_‐based catalysts for CO_2_RR was evaluated in a two‐compartment H‐type cell using a standard three‐electrode configuration with 0.5 M KHCO_3_ aqueous electrolyte (see Figure S4 and the Experimental Section for details). As shown in Figure 3a, the linear sweep voltammetry (LSV) curves demonstrate that M‐SnO_2_ exhibits a significantly higher current density than B‐SnO_2_ under CO_2_RR conditions. As shown in Figure S5a, M‐SnO_2_ requires a 130 mV lower potential than B‐SnO_2_ to reach a current density of −10 mA cm^−2^. Moreover, the comparison of the LSV responses in CO_2_‐ and N_2_‐saturated electrolytes (Figure S5b) further confirms the superior CO_2_RR activity of M‐SnO_2_ through its enhanced current density in the presence of CO_2_ [31]. To gain insights into the electrochemical surface properties, the electrochemical double‐layer capacitance (C_dl_) was determined by the slope of the current capacitive versus scan rate obtained in the non‐Faradaic potential region (Figure S6). M‐SnO_2_ exhibited a larger C_dl_ (0.4 mF cm^−2^) compared to B‐SnO_2_ (0.3 mF cm^−2^), which can be attributed to its higher surface area, more accessible internal pores, and defect‐rich mesoporous structure, indicating a higher electrochemically active surface area. The product selectivity was further investigated via constant‐potential electrolysis at various applied potentials ranging from –0.75 to –1.35 V versus the reversible hydrogen electrode (RHE), with each measurement was performed for 1 h (Figure S7). The liquid‐phase products were analyzed using nuclear magnetic resonance (NMR) spectroscopy (Figure S8). As shown in Figure 3b, the FE for formate reached a maximum at –1.15 V for both catalysts, with M‐SnO_2_ achieving a higher FE of 50.9% compared to 35.0% for B‐SnO_2_, highlighting its enhanced formate selectivity and CO_2_RR efficiency. To evaluate catalytic stability, long‐term electrolysis was performed at –1.15 V. As shown in Figure 3c B‐SnO_2_ exhibited a significant decline in current density over a 12‐hour period. Product analysis at various time intervals (Figure S9) revealed a pronounced decrease in the formate FE, dropping from 34.3% to 15.2%, i.e., a 56% reduction, reflecting the poor electrochemical stability of B‐SnO_2_. In contrast, M‐SnO_2_ demonstrated significantly higher stability, with the formate FE decreasing from 50.9% to 44.3%, corresponding to a 13% reduction, thereby indicating its enhanced durability for CO_2_RR.

To gain further insight into the reaction kinetics at the two distinct SnO_2_ interfaces, electrochemical impedance spectroscopy (EIS) was performed at different potentials in a CO_2_‐saturated 0.5 M KHCO_3_ electrolyte. A few complementary approaches were used to obtain the reaction kinetics from the experimental spectra obtained at different potentials applied to the modified electrodes. First, all the spectra were processed by a simplistic circuit based on a typical solution resistance in series with one R‐CPE unit (Figure S10), and the extracted parameters are summarized in Table S2. Then the charge transfer resistance (R_ct_) was analyzed to assess interfacial charge transport properties. As shown in Figure 3d, under non‐CO_2_RR region (−0.55 to −0.75 V), M‐SnO_2_ displayed a higher R_ct_ than B‐SnO_2_, suggesting a less efficient charge transfer process prior to reaction initiation. This behavior is likely attributable to the enhanced CO_2_ adsorption at the M‐SnO_2_ interface, as evidenced by CO_2_‐TPD (Figure 2a) [32]. The higher CO_2_ enrichment at the catalyst surface may initially hinder charge transfer by increasing the interfacial barrier. Additionally, contact angle measurements revealed that M‐SnO_2_ exhibited a significantly larger contact angle (92°) compared to B‐SnO_2_ (64°), indicating a higher degree of hydrophobicity (Figure S11). The hydrophobic nature of M‐SnO_2_ is expected to facilitate CO_2_ diffusion, thereby enhancing CO_2_ accessibility to active sites [33]. Upon the onset of CO_2_RR (−0.85 to −1.35 V), the R_ct_ of M‐SnO_2_ decreased more rapidly than that of B‐SnO_2_, ultimately reaching a lower value. This trend suggests that once the reaction is initiated, M‐SnO_2_ promotes charge transfer more efficiently, likely due to its mesostructure‐defect synergy, which promotes CO_2_ activation and interfacial reaction kinetics. Second, the pore resistance was investigated as one of the fitted parameters [34, 35, 36]. The results showed only a minor dependence on the applied potential (Figure S13a and Supplementary Note S2). Third, the fitted kinetic parameter (reciprocal of the RC constant; see Figure S13b and Supplementary Note S2) increases as the applied potential becomes more negative (Figure 3e). This potential dependence supports the interpretation as a descriptor of the Faradaic reaction kinetics. At strongly cathodic potentials, M‐SnO_2_ exhibits consistently higher values of this parameter than B‐SnO_2_, indicating faster interfacial reaction kinetics for the mesostructured sample and consistent with its enhanced catalytic performance [37].

### 2.3 Mechanistic Insights

**FIGURE 4:**
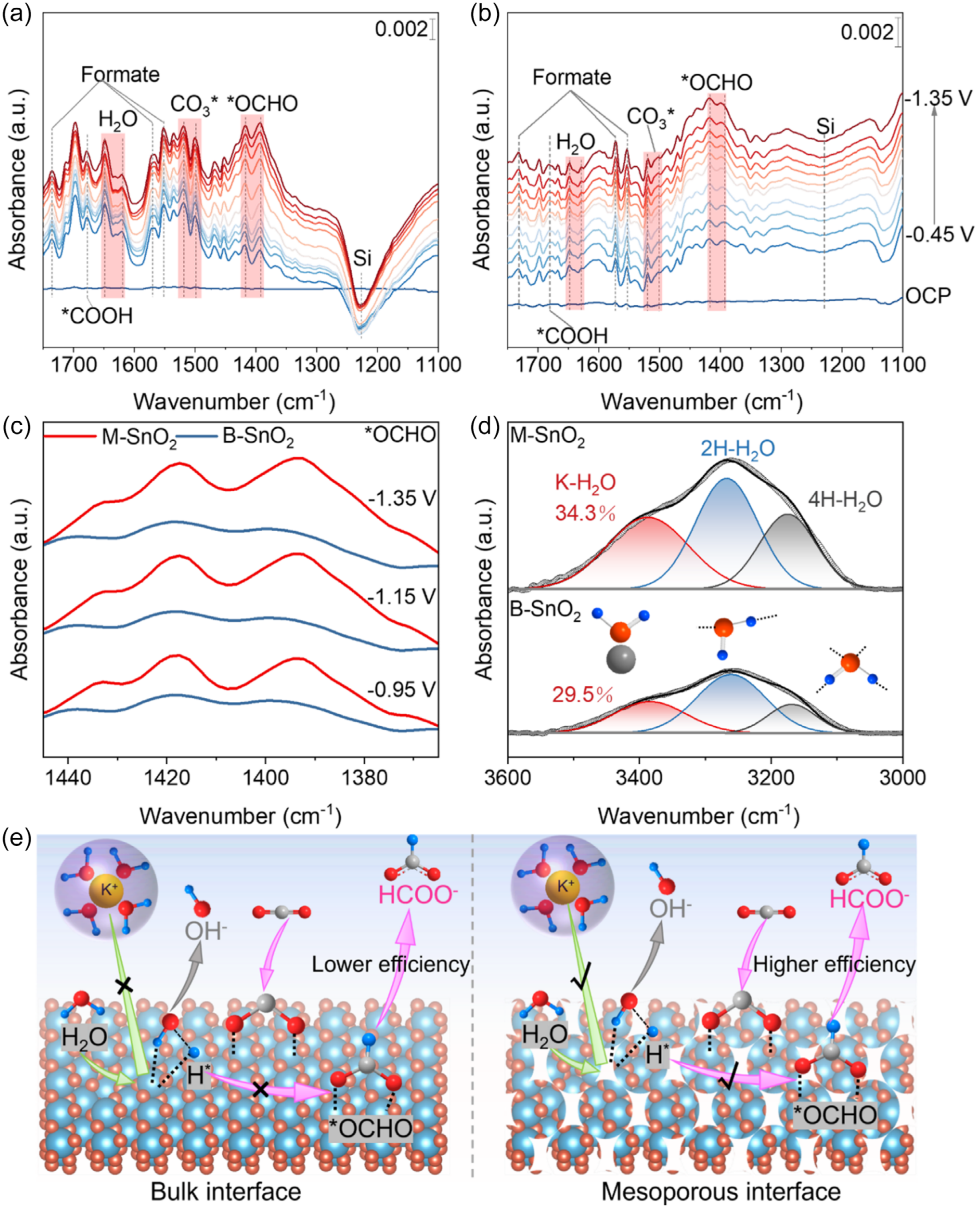
Mechanistic investigations of CO_2_RR on the SnO_2_ interface. (a,b) Potential‐dependent in situ ATR‐SEIRAS spectra of CO_2_RR on M‐SnO_2_ and B‐SnO_2_. The peak at ∼1227 cm^−1^ originates from the Si substrate [38]. (c) Comparison of *OCHO peak absorbance intensity on M‐SnO_2_ and B‐SnO_2_ under different applied potentials. (d) In situ ATR‐SEIRAS spectra of interfacial water at −1.15 V vs. RHE. (e) Schematic illustration of the influence of the mesopore wall structure on CO_2_RR toward formate production.

To elucidate why a mesoporous structure with V_O_ has an enhancing effect on electrocatalytic performance, in situ attenuated total reflection surface‐enhanced infrared absorption spectroscopy (in situ ATR‐SEIRAS) was employed to probe key reaction intermediates during CO_2_RR. Potential‐dependent in situ ATR‐SEIRAS spectra for CO_2_RR on M‐SnO_2_ and B‐SnO_2_ (Figure 4a,b) reveal negative peaks corresponding to intermediate depletion and positive peaks associated with their formation or accumulation. The observed vibrational frequencies and intensities exhibit a strong potential dependence, confirming that the detected signals predominantly originate from the electrode/electrolyte interface.

As shown in Figure 4a,b, both catalysts exhibit characteristic vibrational bands. The bands at ∼1393 and ∼1417 cm^−1^ are attributed to the bidentate *OCHO intermediate, a key species in the formate formation pathway of CO_2_RR [39]. A comparative analysis of intermediates adsorbents on the two catalysts (Figure 4c) reveals two significant differences: (i) the *OCHO signal intensity is substantially higher for M‐SnO_2_, indicating a higher surface coverage of this intermediate (Figure 4c) and (ii) the bands at ∼1500 and ∼1520 cm^−1^, assigned to surface‐bound carbonate species (CO_3_*), are more pronounced for M‐SnO_2_, suggesting an increased degree of carbonate adsorption, which has been correlated with enhanced catalytic activity [40, 41]. Furthermore, the vibrational band at ∼1678 cm^−1^ corresponds to *COOH, a well‐established intermediate for CO formation [42]. Additional formate‐related peaks at ∼1552, ∼1569, and ∼1737 cm^−1^ exhibit stronger intensities on M‐SnO_2_, further supporting the hypothesis that the conditions prevailing inside the mesopores facilitate the accumulation of formate‐related species [43]. To gain deeper insights into reaction kinetics, time‐resolved in situ ATR‐SEIRAS measurements were performed under a constant potential of −1.15 V, with spectra recorded at different time intervals (Figure S14). The spectral features observed under steady‐state conditions closely resemble those in the potential‐dependent measurements, corroborating the stronger *OCHO adsorption on M‐SnO_2_.

In addition to identifying key intermediates, the role of interfacial water in modulating CO_2_RR on M‐SnO_2_ and B‐SnO_2_ was investigated. As shown in Figure S15, the ν(OH) signal of interfacial water appears at 3600–3000 cm^−1^. The ν(OH) intensity is significantly higher for M‐SnO_2_, suggesting enhanced hydrogen‐bonding connectivity at the catalyst‐electrolyte interface. Moreover, the ν(OH) band for M‐SnO_2_ exhibits a pronounced red shift compared to B‐SnO_2_, indicative of a more structured hydrogen‐bonding network [44]. To further investigate this behavior, the interfacial water at the optimal potential of −1.15 V was analyzed (Figure 4d). Gaussian deconvolution of the ν(OH) band revealed three distinct peaks: (i) the low‐wavenumber peak (∼3172 cm^−1^, gray), attributed to 4‐coordinated hydrogen‐bonded water (4H‐H_2_O), (ii) the mid‐wavenumber peak (∼3268 cm^−1^, blue), corresponding to 2‐coordinated hydrogen‐bonded water (2H‐H_2_O), and (iii) the high‐wavenumber peak (∼3390 cm^−1^, red), associated with K^+^‐coordinated water (K‐H_2_O) [45, 46]. The integral area of the K‐H_2_O peak for M‐SnO_2_ is ∼2.6 times that of B‐SnO_2_, indicating much stronger interactions between K^+^ and interfacial water on M‐SnO_2_. Furthermore, the proportion of K‐H_2_O on M‐SnO_2_ (34.3%) is higher than that on B‐SnO_2_ (29.5%), reinforcing the hypothesis that the reaction conditions inside the mesopores with enhanced interfacial interactions promotes CO_2_ activation, which also could be observed at CO_2_‐TPD results (Figure 2a) [47, 48].

Based on these findings, we propose a mechanistic framework for the enhanced formate production on M‐SnO_2_ (Figure 4e). The effects of the mesoporous structure of M‐SnO_2_ promote strong *OCHO adsorption via a higher density of accessible active sites, thereby stabilizing this key intermediate. Additionally, the enrichment of K‐H_2_O at the mesoporous interface facilitates CO_2_ activation and promotes water dissociation, leading to the formation of adsorbed hydrogen species (H*) and the concomitant release of OH^‐^ anions. The generated H* subsequently reacts with adsorbed CO_2_ to form *OCHO, which is further reduced to formate upon accepting an electron.

### 2.4 Catalyst Evolution and Stability in CO_2_RR

**FIGURE 5:**
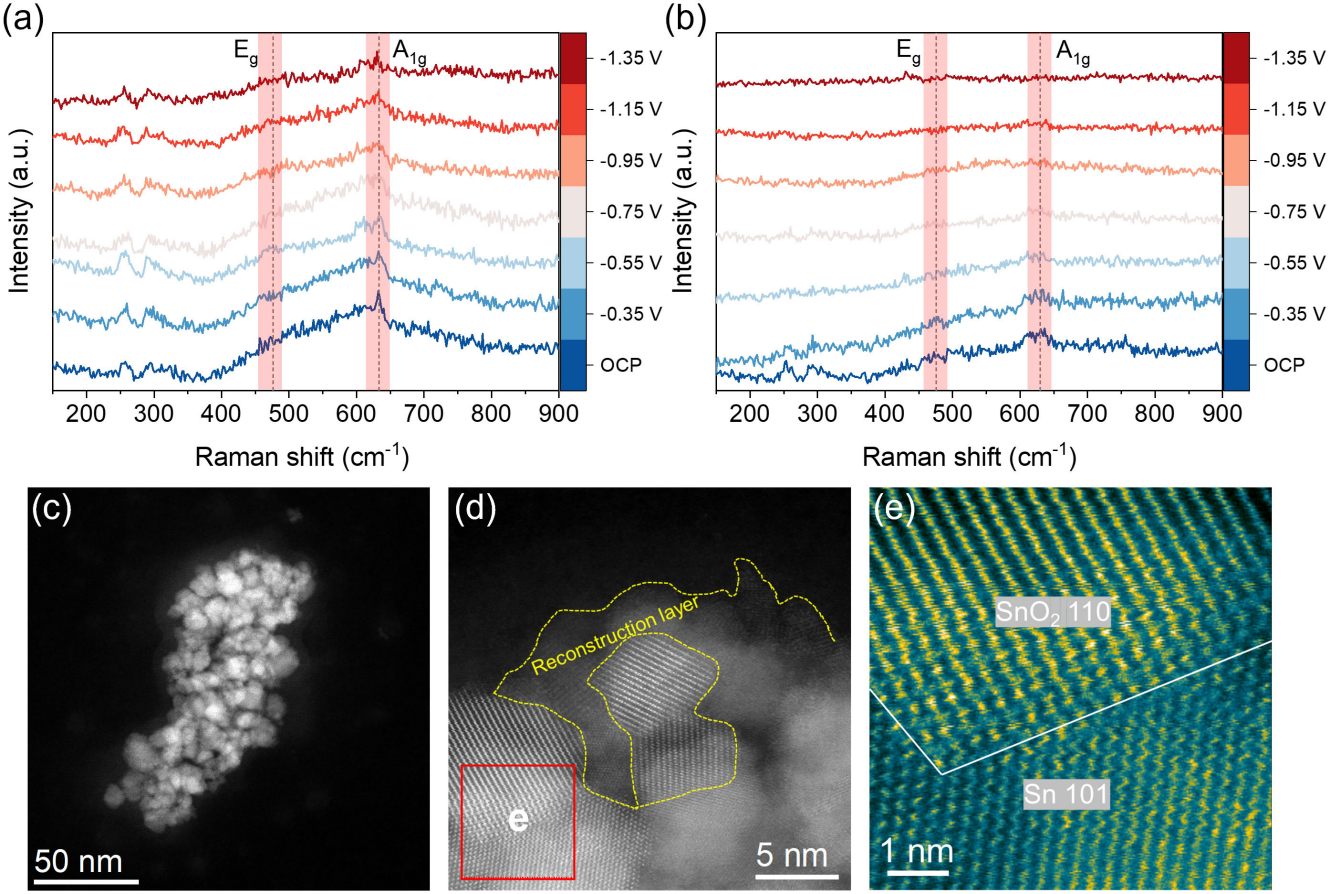
Mechanistic investigation of the dynamic evolution of SnO_2_ catalysts during CO_2_RR. (a,b) Potential‐dependent in situ SERS spectra of M‐SnO_2_ and B‐SnO_2_. (c) HAADF–STEM overview micrograph of M‐SnO_2_ after the stability test. (d) High‐resolution HAADF–STEM micrograph of M‐SnO_2_ after the stability test. (e) Enlarged region from (d), revealing the formation of a SnO_2_/metallic Sn heterogeneous interface.

To investigate the structural evolution of M‐SnO_2_ during electrocatalysis, in situ surface‐enhanced Raman spectroscopy (SERS) was conducted. As shown in Figure 5a,b, the Raman spectra exhibit two characteristic bands at approximately 476 cm^−1^ (E_g_ mode) and 633 cm^−1^ (A_1g_ mode), corresponding to Sn–O vibrational modes and serving as reliable indicators of the Sn oxidation state [49]. With increasingly cathodic potentials, the intensity of these Sn–O bands gradually diminished. Notably, for B‐SnO_2_, the Sn–O signals nearly vanished beyond −0.95 V, indicating extensive reduction of the Sn species. In contrast, the Sn–O bands in M‐SnO_2_ remained clearly detectable even at −1.35 V, suggesting that V_O_ in M‐SnO_2_ plays a stabilizing role in maintaining the Sn oxidation state under reductive conditions, thereby enhancing structural robustness during CO_2_RR. To further verify these observations, X‐ray photoelectron spectroscopy (XPS) was performed to investigate the electronic structure before and after CO_2_RR. As shown in Figure S16, the Sn 3d spectra revealed a smaller reduction in Sn^4+^ for M‐SnO_2_ compared to B‐SnO_2_, indicating greater preservation of the oxidized Sn state. Meanwhile, the O 1s spectra (Figure S17) confirmed that M‐SnO_2_ possessed a higher concentration of V_O_ both before and after CO_2_RR, consistent with its enhanced redox stability. These findings collectively demonstrate that the enhanced oxygen vacancies concentration in M‐SnO_2_ is critical for preserving the SnO_2_ framework and oxidation state under CO_2_RR conditions [50, 51]. Moreover, in situ SERS (Figure S18) revealed the appearance of C–H vibrational bands in the 2800–3000 cm^−1^ range, which are attributed to the *OCHO intermediate, a key species in the CO_2_RR to formate pathway [52, 53]. Importantly, the *OCHO signal on M‐SnO_2_ was noticeably more intense than that observed for B‐SnO_2_, aligning well with the trends observed in ATR‐SEIRAS measurements. These results suggest that the nature of the mesopores in M‐SnO_2_ not only exhibits enhanced structural stability but also promotes the formation of key intermediates, thereby contributing to its better catalytic performance.

To further investigate the structural evolution of M‐SnO_2_, microscopy analysis was performed. As shown in Figure S19, the average particle size increased from 9.1 ± 2.0 nm before CO_2_RR to 10.8 ± 2.4 nm after the reaction, accompanied by a decrease in interparticle mesopores, indicative of particle aggregation and coalescence during CO_2_RR. This was further confirmed by HAADF–STEM micrographs (Figure 5c). HR‐TEM micrographs and corresponding FFT patterns (Figure S20) revealed no significant changes in the SnO_2_ 110 and 101 lattice planes, indicating that the crystalline structure of M‐SnO_2_ remained well‐preserved. However, as shown in Figure 5a, the decreasing intensity of the Sn–O Raman bands at more negative potentials suggests partial reduction of Sn–O to metallic Sn or a lower oxidation state SnO_*x*_. The electrochemical reduction is also visualized by HR‐HAADF–STEM imaging (Figure 5d), showing that part of the SnO_2_ domains is converted into amorphous metallic Sn or SnO_*x*_. Meanwhile, other domains evolve into crystalline metallic Sn, leading to a heterogeneous microstructure featuring distinct Sn/SnO_2_ interfaces (Figure 5e). Consistent features are also observed in additional HAADF–STEM micrographs collected before and after CO_2_RR (Figure S21), indicating that metallic Sn and/or SnO_*x*_ species are mainly generated at the catalyst surface.

In line with these microscopy observations, XRD analysis (Figure S22 and Supplementary Note S4) shows an increase in the coherent crystalline domain size from 6.8 ± 0.1 nm prior to CO_2_RR to 8.5 ± 0.7 nm for the remaining SnO_2_ phase and 36.2 ± 1.4 nm for the metallic Sn phase formed after the reaction, accompanied by partial conversion of SnO_2_ to metallic Sn. Together with the particle growth observed in Figure S19, these results suggest that electrochemical reduction leads to structural coarsening through particle aggregation, coalescence, and phase reconstruction. Similar electrochemically induced reconstruction behavior has been reported previously [54, 55].

### 2.5 The Influence of Oxygen Vacancies on Catalytic Activity

**FIGURE 6:**
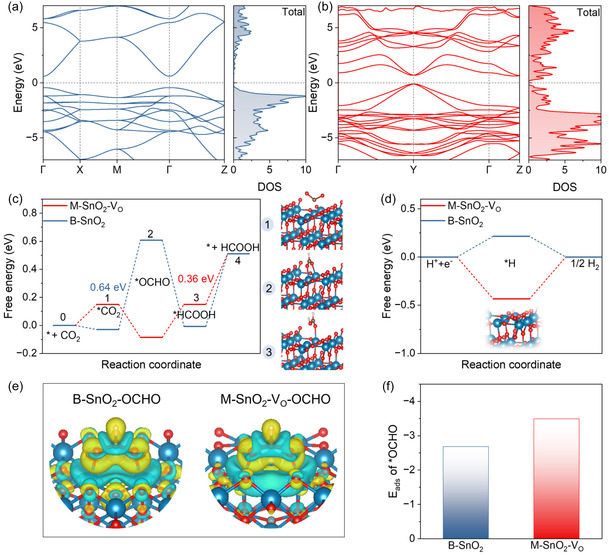
Theoretical investigation of the impact of oxygen vacancies. (a) Calculated DOS and band structures for B‐SnO_2_ and (b) M‐SnO_2_‐V_O_. (c) Free energy diagrams for CO_2_RR to formate on B‐SnO_2_ and M‐SnO_2_‐V_O_, along with the reaction intermediates involved in CO_2_ reduction on M‐SnO_2_‐V_O_. (d) Free energy diagrams for the HER on B‐SnO_2_ and M‐SnO_2_‐V_O_. (e) Differential charge density maps illustrating the adsorption of the *OCHO intermediate on B‐SnO_2_ and M‐SnO_2_‐V_O_ surfaces. (f) Calculated adsorption energies of *OCHO on B‐SnO_2_ and M‐SnO_2_‐V_O_ surfaces.

DFT calculations were employed to investigate the influence of V_O_ on the electronic structure and catalytic performance of M‐SnO_2_ toward CO_2_RR. Figure 6a,b illustrate the band gap structure and density of states (DOS) of the SnO_2_ (B‐SnO_2_) and oxygen‐deficient SnO_2_ (M‐SnO_2_‐V_O_) model. The introduction of V_O_ effectively decreases the band gap and increases the density of electronic states near the Fermi level, which is conducive to improved conductivity. Additionally, the narrower band gap of M‐SnO_2_‐V_O_ than B‐SnO_2_ indicates a higher charge carrier concentration and improved charge transport. The CO_2_RR activity over B‐SnO_2_ and M‐SnO_2_‐V_O_ was subsequently evaluated. As shown in Figure 6c, the rate‐determining step (RDS) over B‐SnO_2_ is the formation of *OCHO, with a large Gibbs free energy barrier (ΔG) of 0.64 eV. In contrast, the Δ*G* for *OCHO formation on M‐SnO_2_‐V_O_ is significantly lower (−0.24 eV), suggesting that the introduction of V_O_ facilitates this step. For the M‐SnO_2_‐V_O_, the RDS shifts to the conversion of *HCOOH to HCOOH, with a reduced Δ*G* of 0.36 eV, suggesting enhanced CO_2_RR activity. Competing hydrogen evolution reaction (HER) was also investigated. As shown in Figure 6d M‐SnO_2_‐V_O_ exhibits weaker *H adsorption (−0.44 eV) compared to B‐SnO_2_ (0.22 eV), indicating that V_O_ partially suppresses HER. Given that *OCHO formation is a key intermediate in formate production [56, 57], the charge density difference analysis in Figure 6e reveals that *OCHO binds to separate Sn atoms in both B‐SnO_2_ and M‐SnO_2_‐V_O_, with the latter exhibiting greater charge accumulation. This redistribution enhances intermediate stabilization, thereby facilitating CO_2_RR. Furthermore, adsorption energy calculations (Figure 6f) confirm that *OCHO binds more strongly on M‐SnO_2_‐V_O_ than on B‐SnO_2_, reinforcing the role of V_O_ in stabilizing reaction intermediates and promoting selective CO_2_RR toward formate production.

## 3 Conclusion

This study demonstrates how mesoporosity and oxygen vacancies synergistically tune the electrocatalytic performance of SnO_2_‐based catalysts for CO_2_RR to formate. Compared to B‐SnO_2_, M‐SnO_2_ achieves a higher FE of 50.9% at −1.15 V vs. RHE and exhibits better long‐term stability. In situ spectroscopic analysis reveals that the mesoporous structure facilitates CO_2_ adsorption and stabilizes *OCHO intermediates, which are essential for selective formate production. DFT calculations further confirm that oxygen vacancies, generated in conjunction with the mesoporous structure, lower the energy barrier for CO_2_RR, thereby enhancing reaction kinetics. The synergy between pore structure and defect sites thus accounts for the higher activity and durability of M‐SnO_2_. Future studies could explore how systematic tuning of pore size, oxygen vacancy concentration, and catalyst‐support interactions influence electron transfer processes, potentially extending these design principles to other metal oxides electrocatalysts for more efficient CO_2_ conversion.

## 4 Experimental Section

### 4.1 Chemicals

The following chemicals were used in this study: hexadecyl trimethyl ammonium bromide (CTAB, ≥99%), tin (IV) chloride pentahydrate (SnCl_4_ · 5H_2_O, 98%), dimethyl sulfoxide (DMSO, ≥99.9%), sodium hydroxide (NaOH, ≥98%), potassium bicarbonate (KHCO_3_, 99.5%), isopropanol (≥99.5%), and a 5 wt% Nafion solution. All were purchased from Merck. Two high‐purity gases, CO_2_ (99.99%) and N_2_ (99.99%), were purchased from Linde gas and used in this study. Absolute ethanol was supplied by Solvevo. Deionized (DI) water was purified using a Millipore Milli‐Q system.

### 4.2 Synthesis of SnO_2_ catalysts

Mesoporous SnO_2_ was synthesized via a surfactant‐templated hydrothermal method from Ge et al. [26] with some modifications. Briefly, 15 mmol CTAB was dissolved in 200 mL DI water under vigorous stirring for 1 h. Subsequently, 12 mmol of NaOH was added to the solution, and stirring was continued for 30 min. A separate aqueous solution of SnCl_4_ · 5H_2_O (7.5 mmol in 10 mL DI water) was then introduced dropwise into the mixture, followed by stirring for 2 h at room temperature. The resulting homogeneous solution was transferred into a Teflon bottle and hydrothermally treated at 100°C for 48 h. The precipitate was collected by centrifugation, washed thoroughly with DI water and ethanol, and dried overnight at 100°C. Finally, the product was calcined at 500°C for 2 h (ramp rate: 5°C/min) to remove the surfactant template and crystallize the SnO_2_ framework. For comparison, bulk SnO_2_ was prepared using an identical procedure but without CTAB to eliminate mesopore formation.

### 4.3 Characterizations

X‐ray diffractometry (XRD) was conducted on a Panalytical X’Pert Pro diffractometer equipped with Cu Kα radiation (*λ* = 0.15406 nm), operating at 40 kV and 40 mA. Data were collected over a 2θ range of 20°–80° with a step size of 0.05°. N_2_ physisorption measurements were performed at −196°C using a Micromeritics ASAP 2020 analyzer. Prior to analysis, the samples were degassed at 100°C under a vacuum for 4 h to remove surface contaminants. The specific surface area was determined by the Brunauer–Emmett–Teller (BET) method, utilizing adsorption data in the relative pressure range (P/P_0_) of 0.07–0.18. The surface morphologies of the SnO_2_ catalysts were examined using a scanning electron microscope (Sigma 300, Zeiss). Images were acquired in in‐lens mode with an accelerating voltage of 5 kV. Transmission electron microscopy (TEM) and high‐angle annular dark‐field scanning TEM (HAADF–STEM) were conducted using an FEI Tecnai G2 microscope (200 kV) and an FEI Titan G2 microscope (300 kV), respectively. Energy‐dispersive X‐ray spectroscopy (EDS) was employed for elemental analysis. Temperature‐programmed desorption of CO_2_ (CO_2_‐TPD) was carried out on a Micromeritics AutoChem II 2920 analyzer. Approximately 200 mg of the sample was loaded into a quartz reactor and pretreated at 500°C under He flow for 1 h to remove adsorbed species. After cooling to 50°C, the sample was exposed to a 10% CO_2_/He mixture for 1 h, followed by He purging for an additional hour to eliminate weakly adsorbed CO_2_. The TPD profile was recorded by heating the sample to 350°C under He flow, with desorbed gases monitored using a thermal conductivity detector (TCD). XPS spectra were recorded using a Scienta‐200 hemispherical analyzer with a monochromatized Al Kα source (1486.6 eV). All measurements were conducted under ultra high vacuum (UHV) conditions with a base pressure below 1 × 10^−9^ mbar.

### 4.4 Electrochemical Measurements

All electrochemical measurements were performed using a BioLogic SP‐200 workstation in an H‐cell separated by a Nafion 115 membrane, employing a three‐electrode system with an Ag/AgCl (saturated KCl) reference electrode and Pt plate counter electrode. The working electrode was prepared by ultrasonically dispersing 7 mg catalyst in a mixture of 450 μL DI water, 500 μL isopropanol, and 50 μL 5 wt% Nafion solution for 1 h, followed by drop‐casting 200 μL of the homogeneous ink onto 1 cm^2^ carbon paper and drying. For LSV measurements (10 mV/s scan rate), the 0.5 M KHCO_3_ electrolyte was presaturated by purging CO_2_ or N_2_ for at least 30 min. Chronoamperometry (CA) tests were conducted under constant potential with continuous CO_2_ flow in 0.5 M KHCO_3_ (20 mL/min), while EIS measurements were performed from 10 mHz to 100 kHz (10 mV amplitude) in CO_2_‐saturated electrolyte at various potentials. All potentials were referred to Ag/AgCl and converted to RHE using the following Nernst equation without iR compensation:

(1) $$E_{\left(\mathrm{vs}.\mathrm{RHE}\right)}=E_{\left(\mathrm{vs}.\mathrm{Ag}/\mathrm{AgCl}\right)}+0.059\mathrm{pH}+0.197$$

### 4.5 Products analysis

The formate was analyzed by ^1^H‐NMR (500 MHz, Bruker). Dimethyl sulfoxide in D_2_O was used as an internal standard to quantify the products. For each measurement, 500 μL of the electrolyte and 100 μL 0.2 wt% DMSO in D_2_O were mixed. The measurements were performed with water suppression. The FE of formate can be calculated by using the following equation:

(2) $$FE_{\left(\text{formate}\right)}=\frac{n\times N\times F}{j\times t}\times 100\%$$

where *n* is the number of moles of formate produced in the cathodic electrolyte, *N* is 2, *F* is 96 485 C mol^−1^, *t* is the reaction time, and *j* is the current density.

### 4.6 In situ SERS

For electrode preparation, the catalyst ink was mixed with a commercial Au SERS nanoparticle suspension (0.05 mg mL^−1^, ∼100 nm in diameter) and sonicated for 1 h to ensure uniform dispersion. The homogeneous mixture was then drop‐cast stepwise onto a glassy carbon electrode and dried at room temperature. The measurements were conducted using a confocal Raman spectroscope (Renishaw Invia Raman microscope) equipped with a 532 nm laser, operated at approximately 50% power, an L50X objective lens, and a charge‐coupled device (CCD) detector. A homemade three‐electrode in situ Raman cell was employed, featuring a Pt wire counter electrode and an Ag/AgCl reference electrode (saturated KCl solution). Electrolysis was performed using a CHI 760 potentiostat in a CO_2_‐saturated 0.5 M KHCO_3_ aqueous solution, which was continuously supplied via a peristaltic pump. Each Raman spectrum was recorded with an exposure time of 10s and five accumulations to enhance signal quality. For a typical measurement, chronoamperometry was used to maintain a stable current response. Raman spectra were collected at different applied potentials after 2 min of electrolysis, ensuring a steady‐state condition before acquisition.

### 4.7 In Situ ATR‐SEIRAS

The measurements were done using a Bruker Vertex 70 Fourier transform infrared spectrometer, equipped with a liquid nitrogen‐cooled mercury cadmium tellurium (MCT) detector and attenuated total reflection accessories (Pike, Vee Max III). The experiment was conducted in a 0.5 M KHCO_3_ solution saturated with CO_2_, with CO_2_ being continuously injected throughout the experiment. Here, platinum foil and Ag/AgCl were used as counter and reference electrodes, respectively. For the working electrode, the catalyst inks were drop‐cast onto a silicon crystal coated with an Au film. The experimental conditions are as follows: a resolution of 4 cm^−1^ and each test scanning duration of around 60 s.

### 4.8 Density Functional Theory Calculations

DFT calculations were performed using the Vienna Ab initio Simulation Package (VASP) [58, 59]. The electron‐ion interactions were described by the projector‐augmented wave (PAW) method [60], while the exchange‐correlation effects were treated within the generalized gradient approximation (GGA) using the Perdew–Burke–Ernzerhof (PBE) functional [61]. A plane‐wave cutoff energy of 450 eV was employed for all spin‐polarized calculations. The SnO_2_ 110 surface was modeled using a (2 × 2) supercell constructed from the optimized bulk rutile‐phase SnO_2_ (space group: P42/mnm). For the SnO_2__Vo, oxygen atoms are selectively removed to introduce vacancies. During geometry optimization, the top two atomic layers were allowed to relax, while the bottom layers were fixed to mimic the bulk‐like environment. For surface calculations, the Brillouin zone was sampled using a (5 × 3 × 1) Monkhorst–Pack k‐point grid [62], whereas a (3 × 3 × 1) grid was used for gas‐phase species. A Gaussian smearing width of 0.1 eV was applied for surface calculations, while a finer smearing of 0.01 eV was used for isolated molecules. Electronic convergence was achieved with a tolerance of 10^−5^ eV, and ionic relaxation was performed until the maximum force on each atom fell below 0.03 eV Å^−1^. To ensure the most stable adsorption configuration, multiple initial geometries of intermediate species were tested, and the lowest‐energy structure was selected for further analysis. The Gibbs free energy change (ΔG) of each elementary step is calculated based on the computational hydrogen electrode (CHE) model proposed by Nørskov et al. [63]. According to the CHE model, Δ*G* can be defined as:

(3) $$\Delta G=\Delta E+\Delta ZPE+\int C_{p}\Delta T-T\Delta S$$

where Δ*E* is the electronic energy difference, Δ*ZPE* denotes the zero‐point energy correction, and Δ*S* represents the entropy change. The enthalpic contribution (∫*C*_*p*_ dT) accounts for heat capacity effects, and T = 298.15 K is the reference temperature.

The electrochemical reduction of CO_2_ to formic acid (HCOOH) proceeds via three sequential steps:



(4)

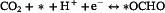






(5)

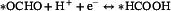






(6)

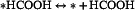




The projected density of states (PDOS) for each atomic species was computed using the optimized geometries, with a denser Monkhorst–Pack k‐point grid of (10 × 6 × 1) for the SnO_2_ (110) surface and (9 × 9 × 1) for bulk‐like comparisons in non‐self‐consistent calculations. To elucidate the electronic interaction between *OCHO intermediate and the SnO_2_ surface, a charge density difference analysis was performed.

## 5 Supporting Information

Additional supporting information can be found online in the Supporting Information section. **Supporting Fig. S1**: (a, b) HAADF−STEM micrograph of M‐SnO_2_ with different magnifications. (c, d) the corresponding FFT patterns from different areas. **Supporting Fig. S2**: CO_2_‐TPD profiles of M‐SnO_2_ and B‐SnO_2_, with TCD signal intensity normalized to the specific surface area (SSA). **Supporting Fig. S3**: (a) UV‐visible absorption spectra of M‐SnO_2_ and B‐SnO_2_. (b) Tauc plots used to determine band gaps. (c) EPR spectra shows the intensity of the signal associated with oxygen vacancies. **Supporting Fig. S4**: Set up for the evaluation of electrocatalytic CO_2_RR performance. **Supporting Fig. S5**: (a) Potentials required to reach ‐10 mA cm^‐2^ for B‐SnO_2_ and M‐SnO_2_. (b) LSV curves of M‐SnO_2_ under two different conditions: CO_2_ and N_2_ saturated 0.5 M KHCO_3_. **Supporting Fig. S6**: CV curves recorded at different scan rates for (a) M‐SnO_2_ and (b) B‐SnO_2_ in 0.5 M CO_2_‐saturated KHCO_3_. (c) Charging current density differences (Δj/2) plotted against scan rates for M‐SnO_2_ and B‐SnO_2_. **Supporting Fig. S7**: Constant potential electrolysis of (a) B‐SnO_2_ and (b) M‐SnO_2_ at each applied potential for 1 h in CO_2_‐saturated 0.5 M KHCO_3_. **Supporting Fig. S8**: NMR spectra of the electrolyte obtained after one hour of electrolysis using (a) B‐SnO_2_ and (b) M‐SnO_2_. **Supporting Fig. S9**: NMR spectra of the electrolyte collected after different durations of the CO_2_RR stability test using (a) M‐SnO_2_ and (b) B‐SnO_2_ at a constant potential of ‐1.15 V. **Supporting Fig. S10**: (a) The EIS fitting circuit includes three components: R_s_, representing the solution resistance; CPE1, denoting the constant phase element; and R_ct_, indicating the interfacial charge transfer resistance. EIS measurements at various applied potentials of (b) M‐SnO_2_ and (c) B‐SnO_2_ in CO_2_‐saturated 0.5 M KHCO_3_, with solid lines representing fitted curves. **Supporting Fig. S11**: Contact angles of M‐SnO_2_ and B‐SnO_2_. **Supporting Fig. S12**: The impedance spectra of electrode modified by B‐SnO_2_ at ‐0.95 V (a) and ‐1.15 V (b) Insets: equivalent circuits utilized for fitting (as an example of how the fitting was performed). **Supporting Fig. S13**: The potential dependences of pore resistance (a) and RC (b) estimated from the circuit with finite length De Levie element (inset of Figure S12a). **Supporting Fig. S14**: Time‐dependent in situ ATR‐SEIRAS of (a) M‐SnO_2_ and (b) B‐SnO_2_. **Supporting Fig. S15**: In situ ATR‐SEIRAS of interfacial water over M‐SnO_2_ and B‐SnO_2_ under different applied potentials. **Supporting Fig. S16**: Sn 3d XPS spectra of (a) B‐SnO_2_ and (b) M‐SnO_2_ before and after CO_2_RR. (c) The proportion of Sn^2+^/Sn^4+^ of B‐SnO_2_ and M‐SnO_2_ after CO_2_RR (1.15 V vs. RHE, 12 h). **Supporting Fig. S17**: O 1s XPS spectra of (a) B‐SnO_2_ and (b) M‐SnO_2_ before and after CO_2_RR. **Supporting Fig. S18**: In situ SERS of C‐H on the (a) M‐SnO_2_ and (b) B‐SnO_2_. **Supporting Fig. S19**: HAADF‐STEM micrographs of M‐SnO_2_ before and after CO_2_RR, accompanied by an analysis of particle size evolution. **Supporting Fig. S20**: HR‐TEM micrographs of M‐SnO_2_ (a) before and (b) after CO_2_RR and the corresponding FFT patterns. **Supporting Fig. S21**: HR HAADF‐STEM micrographs of (a) M‐SnO_2_ and (b) corresponding inverse fast Fourier transform (IFFT) patterns from (a). **Supporting Fig. S22**: (a) X‐ray diffractograms of M‐SnO_2_ before and after CO_2_RR. (b) Comparation of crystallite size of M‐SnO_2_ before and after CO_2_RR. **Supporting Table S1**: EDS mapping elements summary of M‐SnO_2_. **Supporting Table S2**: The parameters of impedance spectra fitting by R‐CPE circuit. **Supporting Table S3**: The parameters of impedance spectra fitting by circuit with De Levie element. **Supporting Note S1**: In M‐SnO_2_, VO are likely introduced during the formation of mesopores through the nanoparticles self‐assembly, where the exposure of low‐coordinated atoms reduces the formation energy of VO [1, 2]. In addition, the formation of numerous grain boundaries, likely originating from the aggregation of nanosized particles, implies the presence of exposed low‐coordination surface sites that are prone to VO formation, as evidenced by the STEM micrograph (Figure S1) [3, 5]. Since VO can act as active sites in electrocatalytic reactions, their presence is particularly relevant to CO2RR performance. Consistently, the enhanced UV absorption of M‐SnO_2_ over the entire wavelength range may originate from a broad distribution of localized states within the band gap, likely associated with structural defects such as oxygen vacancies (Figure S3a) [6, 7]. As determined by Tauc plot method, the band gap energy of M‐SnO_2_ (3.49 eV) is lower than that of B‐SnO_2_ (3.98 eV), implying enhanced electric conductivity (Figure S3b). Electron paramagnetic resonance (EPR) spectroscopy (Figure S3c) further substantiates these findings. Both samples display an EPR signal at g ≈ 2.003, which could be associated with oxygen vacancy related paramagnetic centers. The spin intensity of M‐SnO_2_ is about 1.1 times higher than that of B‐SnO_2_, suggesting an increased population of oxygen vacancy related defects. This is also further confirmed by the X‐ray photoelectron spectroscopy (XPS) results in Figure S17. As oxygen vacancies provide electron‐rich sites that facilitate CO_2_ activation, the increased defect density in M‐SnO_2_ is expected to enhance catalytic performance and help stabilize the oxidation state of SnO_2_ [5, 8]. **Supporting Note S2**: An equivalent circuit developed for reactive and conductive cylindrical pores, so called De Levie pores, was utilized [9, 10]. Here, the equivalent circuit consists of solution resistance in series with element containing finite De Levie element in parallel with the film capacitor (inset in Figure S12a). Good fit was obtained only at the frequencies above 10 Hz (Table S3). Due to the unknown number of pores, the kinetics of the Faradaic process can be represented by resistor–capacitor (RC) constant instead of single fitted parameter [11]. The dependence of RC constant on the potential showed minor effect of mesoporosity except the onset potential of the general Faradaic reactions. The use of the equivalent circuit with element proposed by Paasch [12] (inset of Figure S12b) enabled to fit a whole spectrum in a wide frequency region. **Supporting Note 3**: To investigate the electronic structure changes before and after CO_2_RR, XPS measurements were performed on the electrodes. As shown in Figure S17, the concentration of oxygen vacancies in M‐SnO_2_ is higher than that in B‐SnO_2_, which is consistent with the EPR results in Figure 2f. After CO_2_RR, partial reduction of Sn^4+^ occurred along with structural reconstruction, leading to the formation of additional oxygen vacancies. **Supporting Note 4**: As shown in Figure S22, diffraction peaks corresponding to metallic Sn appear after CO_2_RR, matching well with the standard Sn XRD pattern (PDF #04‐0673). This result indicates that part of M‐SnO_2_ was reduced to metallic Sn, which is consistent with the STEM observations of M‐SnO_2_ after CO_2_RR. To estimate the crystalline domain size, XRD peaks in the 2θ range of 30°–36° were fitted using Gaussian functions. The associated error bars were derived from the standard errors of the full width at half maximum (FWHM) obtained from the fitting. The coherent crystalline domain size was then calculated using the Scherrer equation as follows:

$$D=\frac{K\lambda }{\beta \cos\theta }$$

Where K is dimensionless shape factor (0.9), *λ* is the X‐ray wavelength, *β* is the FWHM of the fitted planes, and *θ* is the corresponding diffraction angle.

## Funding

This study was supported by Energimyndigheten (2022‐00909), National Natural Science Foundation of China (22278012), Knut och Alice Wallenbergs Stiftelse (2022.0034), Vetenskapsrådet (2020‐04538), Swedish Government Strategic Research Area in Materials Science on Functional Materials at Linkoping University (2009‐00971).

## Conflicts of Interest

The authors declare no conflicts of interest.

## Supporting information

Supplementary Material

## Data Availability

The data that support the findings of this study are available from the corresponding author upon reasonable request.
