# Supplementary material for: Synergistic Effects of Mesoporous Structure and Oxygen Vacancies in SnO2 for Enhanced CO2 Electroreduction
Source: Small Sci. 2026 Mar 30;6(4):e70268. doi: 10.1002/smsc.70268 (PMC13154916; doi:10.1002/smsc.70268)
Supplement: Supplementary file 1 — Supplementary Material [file SMSC-6-e70268-s001.pdf]

Supplementary Information

Synergistic Effects of Mesoporous Structure and Oxygen Vacancies in SnO_2 for Enhanced CO_2 Electroreduction

Yuguo Zhao,[1]* Shoushuang Huang,[2] Yong Yan,[3] Robert Boyd,[1] Zesheng Liu,[4] Mats Fahlman,[4] Mikhail Vagin,[4] Magnus Odén,[1] and Emma M. Björk[1]

[1] Nanostructured Materials, Department of Physics, Chemistry and Biology (IFM), Linköping University, 58183 Linköping, Sweden.
[2] Division of Molecular Surface Physics & Nanoscience, Department of Physics, Chemistry and Biology (IFM), Linköping University, 58183 Linköping, Sweden.
[3] State Key Laboratory of Materials Low-Carbon Recycling, Center of Excellence for Environmental Safety and Biological Effects, Department of Chemistry, College of Chemistry and Life Science, Beijing University of Technology, Beijing 100124, P. R. China.
[4] Laboratory of Organic Electronics, Department of Science and Technology, Linköping University, 60174 Norrköping, Sweden.

Corresponding author: Yuguo Zhao: yuguo.zhao@liu.se

Table of contents

1. Supplementary Figures 4

   Figure S1. (a, b) HAADF−STEM micrograph of M-SnO_2 with different magnifications. (c, d) the corresponding FFT patterns from different areas. 4

   Figure S2. CO_2-TPD profiles of M-SnO_2 and B-SnO_2, with TCD signal intensity normalized to the specific surface area (SSA). 5

   Figure S3. (a) UV-visible absorption spectra of M-SnO_2 and B-SnO_2. (b) Tauc plots used to determine band gaps. (c) EPR spectra shows the intensity of the signal associated with oxygen vacancies. 6

   Figure S4. Set up for the evaluation of electrocatalytic CO_2RR performance. 7

   Figure S5. (a) Potentials required to reach -10 mA cm^{-2} for B-SnO_2 and M-SnO_2. (b) LSV curves of M-SnO_2 under two different conditions: CO_2 and N_2 saturated 0.5 M $KHCO_3$. 8

   Figure S6. CV curves recorded at different scan rates for (a) M-SnO_2 and (b) B-SnO_2 in 0.5 M CO_2-saturated $KHCO_3$. (c) Charging current density differences ($\Delta j/2$) plotted against scan rates for M-SnO_2 and B-SnO_2. 8

   Figure S7. Constant potential electrolysis of (a) B-SnO_2 and (b) M-SnO_2 at each applied potential for one hour in CO_2-saturated 0.5 M $KHCO_3$. 9

   Figure S8. NMR spectra of the electrolyte obtained after one hour of electrolysis using (a) B-SnO_2 and (b) M-SnO_2. ... 9

   Figure S9. NMR spectra of the electrolyte collected after different durations of the CO_2RR stability test using (a) M-SnO_2 and (b) B-SnO_2 at a constant potential of -1.15 V. 10

   Figure S10. (a) The EIS fitting circuit includes three components: R_s, representing the solution resistance; CPE1, denoting the constant phase element; and R_{ct}, indicating the interfacial charge transfer resistance. EIS measurements at various applied potentials of (b) M-SnO_2 and (c) B-SnO_2 in CO_2-saturated 0.5 M $KHCO_3$, with solid lines representing fitted curves. 10

   Figure S11. Contact angles of M-SnO_2 and B-SnO_2. 11

   Figure S12. The impedance spectra of electrode modified by B-SnO_2 at -0.95 V (a) and -1.15 V (b) Insets: equivalent circuits utilized for fitting (as an example of how the fitting was performed). 12

   Figure S13. The potential dependences of pore resistance (a) and RC (b) estimated from the circuit with finite length De Levie element (inset of Figure S12a). 12

   Figure S14. Time-dependent in situ ATR-SEIRAS of (a) M-SnO_2 and (b) B-SnO_2. 13

   Figure S15. In situ ATR-SEIRAS of interfacial water over M-SnO_2 and B-SnO_2 under different applied potentials..... 14

   Figure S16. 15

   Figure S17. O 1s XPS spectra of (a) B-SnO_2 and (b) M-SnO_2 before and after CO_2RR. 16

   Figure S18. In situ SERS of C-H on the (a) M-SnO_2 and (b) B-SnO_2. 17

   Figure S19. HAADF-STEM micrographs of M-SnO_2 before and after CO_2RR, accompanied by an analysis of particle size evolution. 18

   Figure S20. HR-TEM micrographs of M-SnO_2 (a) before and (b) after CO_2RR and the corresponding FFT patterns... 18

   Figure S21. HR HAADF-STEM micrographs of (a) M-SnO_2 and (b) corresponding inverse fast Fourier transform (IFFT) patterns from (a). 19

   Figure S22. (a) X-ray diffractograms of M-SnO_2 before and after CO_2RR. (b) Comparation of crystallite size of M-SnO_2 before and after CO_2RR 19

2. Supplementary Tables 20

   Table S1. EDS mapping elements summary of M-SnO_2 20

   Table S2. The parameters of impedance spectra fitting by R-CPE circuit 20

Table S3. The parameters of impedance spectra fitting by circuit with De Levie element. 21
3. References 22

1. Supplementary Figures

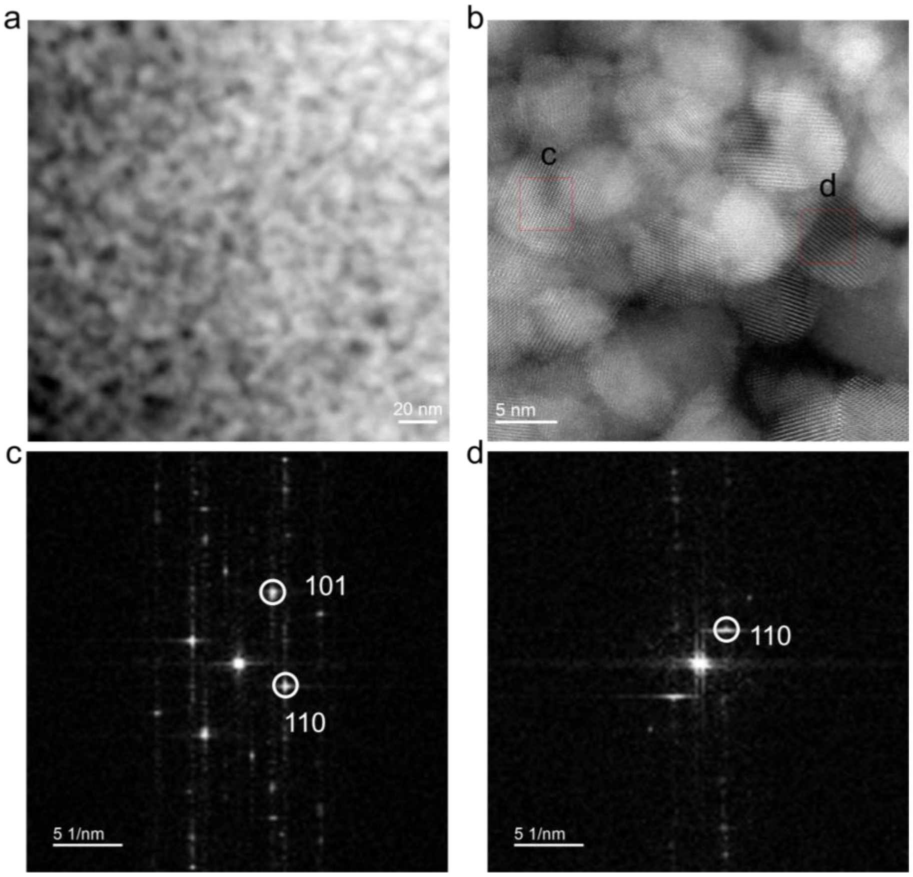

Figure S1. (a, b) HAADF−STEM micrograph of M-SnO_2 with different magnifications. (c, d) the corresponding FFT patterns from different areas.

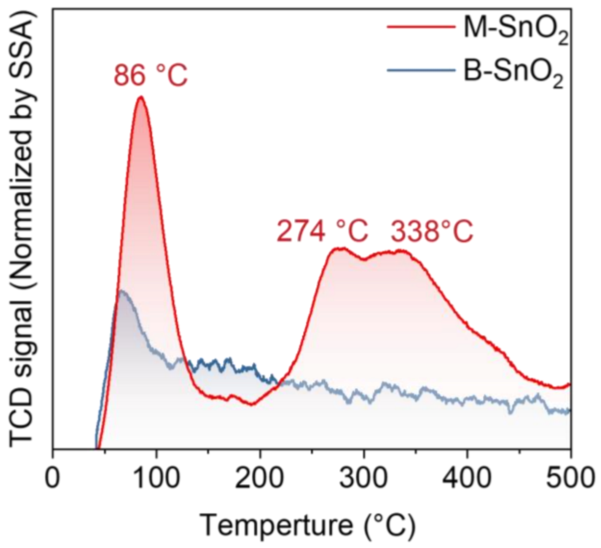

Figure S2. CO_2-TPD profiles of M-SnO_2 and B-SnO_2, with TCD signal intensity normalized to the specific surface area (SSA).

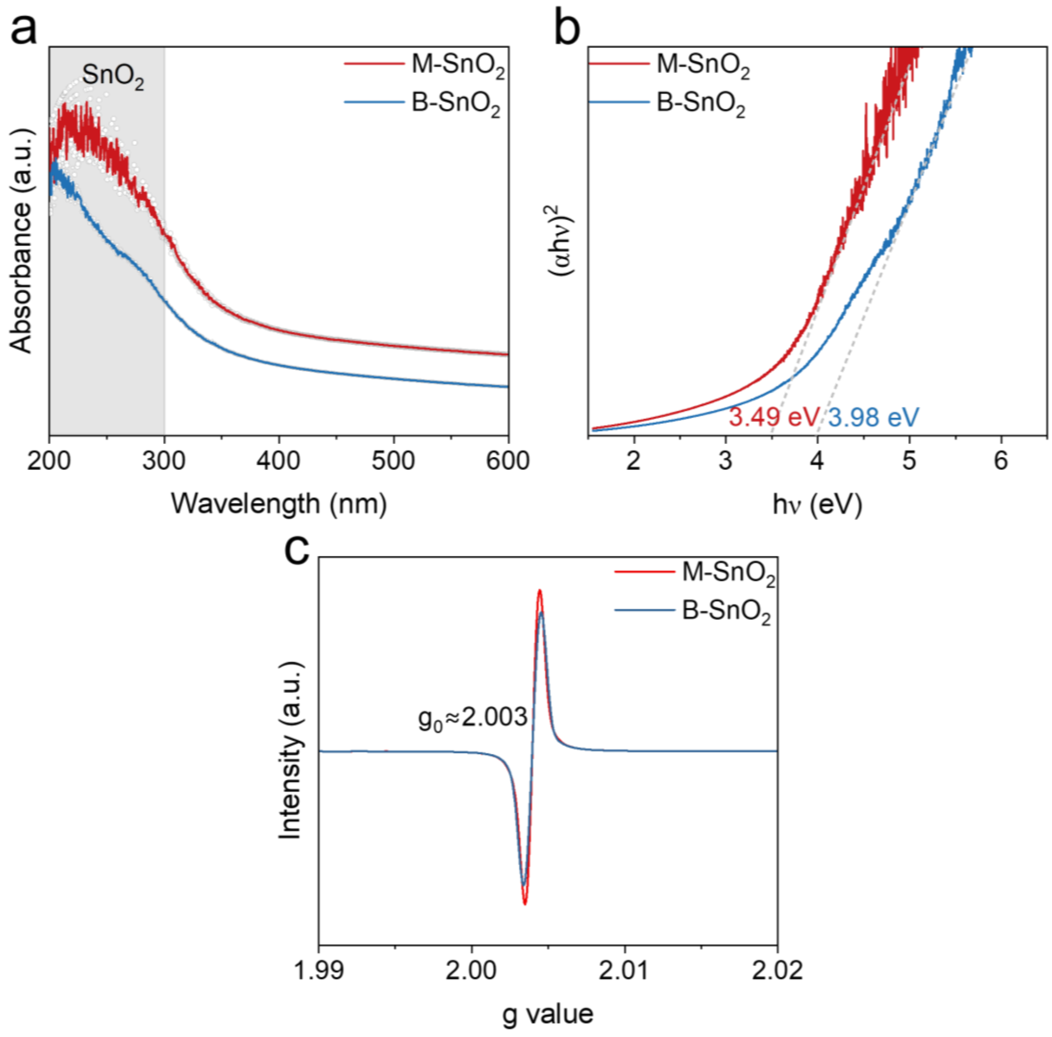

Figure S3. (a) UV-visible absorption spectra of M-SnO_2 and B-SnO_2. (b) Tauc plots used to determine band gaps. (c) EPR spectra shows the intensity of the signal associated with oxygen vacancies.

Supplementary Note 1: In M-SnO_2, V_O are likely introduced during the formation of mesopores through the nanoparticles self-assembly, where the exposure of low-coordinated atoms reduces the formation energy of V_O.[1, 2] In addition, the formation of numerous grain boundaries, likely originating from the aggregation of nanosized particles, implies the presence of exposed low-coordination surface sites that are prone to V_O formation, as evidenced by the STEM micrograph (Figure S1). [3-5] Since V_O can act as active sites in electrocatalytic reactions, their presence is particularly relevant to CO_2RR performance. Consistently, the enhanced UV absorption of M-SnO_2 over the entire wavelength range may originate from a broad distribution of localized states within the band gap, likely associated with structural defects such as oxygen vacancies (Figure S3a).[6, 7] As determined by Tauc plot method, the band gap energy of M-SnO_2 (3.49 eV) is lower than that of B-SnO_2 (3.98 eV), implying enhanced electric conductivity (Figure S3b). Electron paramagnetic resonance (EPR) spectroscopy (Figure S3c) further substantiates these findings. Both samples display an EPR signal at $g \approx 2.003$, which could be associated with oxygen vacancy related paramagnetic centers. The spin intensity of M-SnO_2 is about 1.1 times higher than that of B-SnO_2, suggesting an increased population of oxygen vacancy related defects. This is also further confirmed by the X-ray photoelectron

spectroscopy (XPS) results in Figure S17. As oxygen vacancies provide electron-rich sites that facilitate CO_2 activation, the increased defect density in M-SnO_2 is expected to enhance catalytic performance and help stabilize the oxidation state of SnO_2.[5, 8]

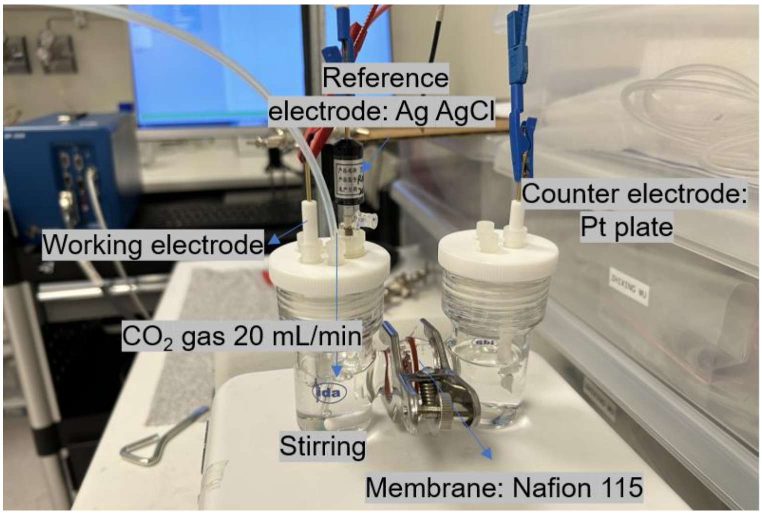

Figure S4. Set up for the evaluation of electrocatalytic CO_2RR performance.

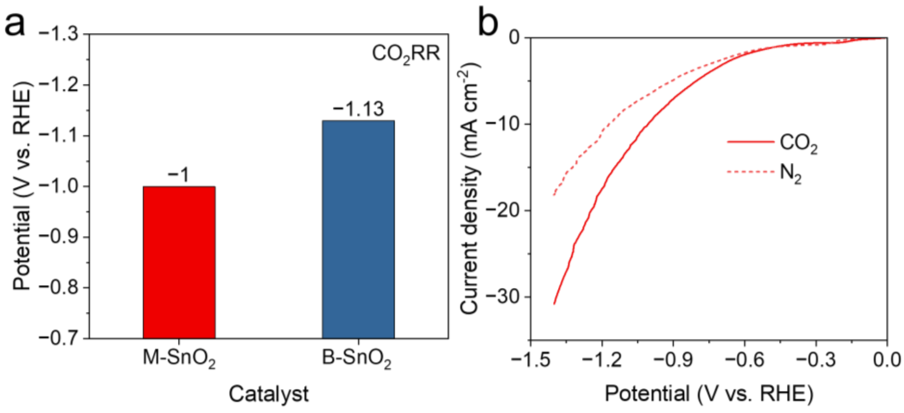

Figure S5. (a) Potentials required to reach -10 mA cm^{-2} for B-SnO_2 and M-SnO_2. (b) LSV curves of M-SnO_2 under two different conditions: CO_2 and N_2 saturated 0.5 M $KHCO_3$.

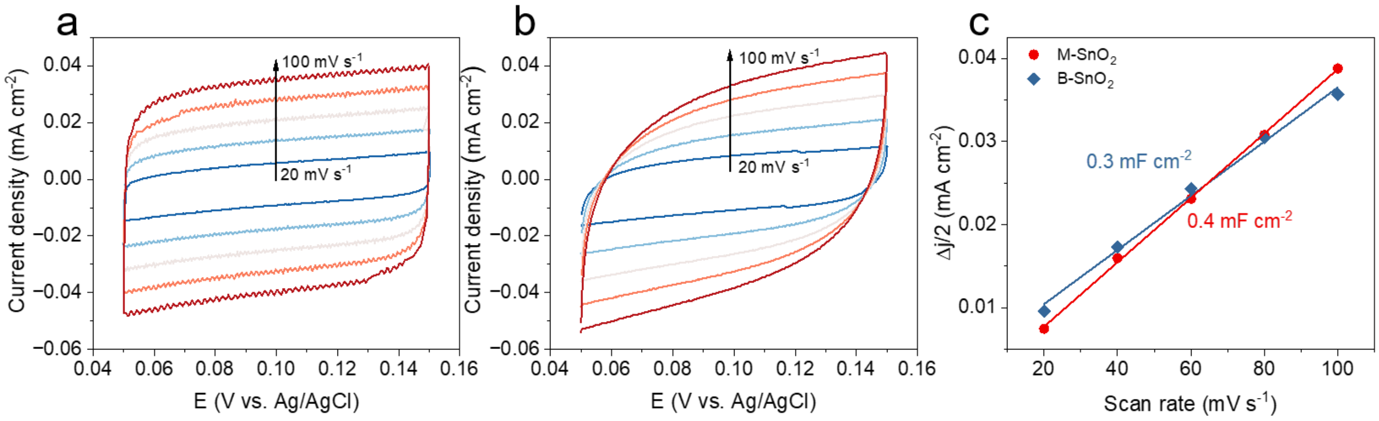

Figure S6. CV curves recorded at different scan rates for (a) M-SnO_2 and (b) B-SnO_2 in 0.5 M CO_2-saturated $KHCO_3$. (c) Charging current density differences (Δj/2) plotted against scan rates for M-SnO_2 and B-SnO_2.

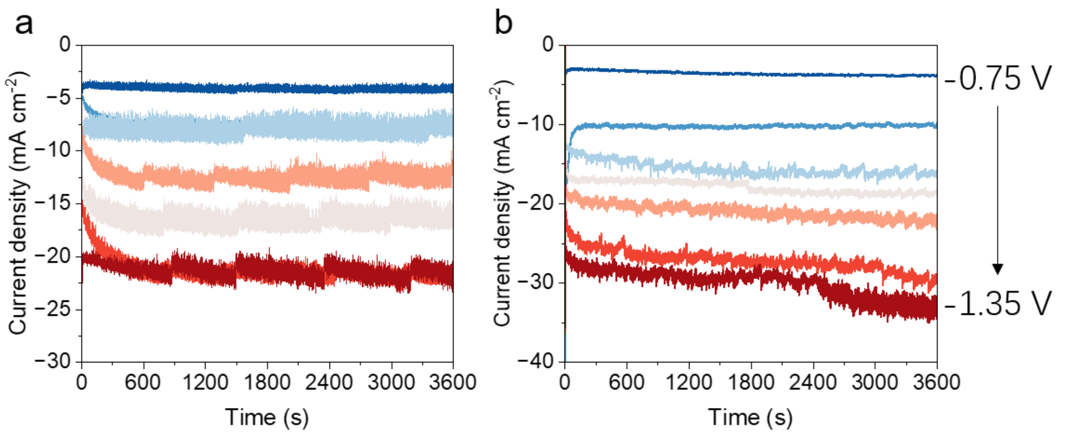

Figure S7. Constant potential electrolysis of (a) B-SnO_2 and (b) M-SnO_2 at each applied potential for one hour in CO_2-saturated 0.5 M $KHCO_3$.

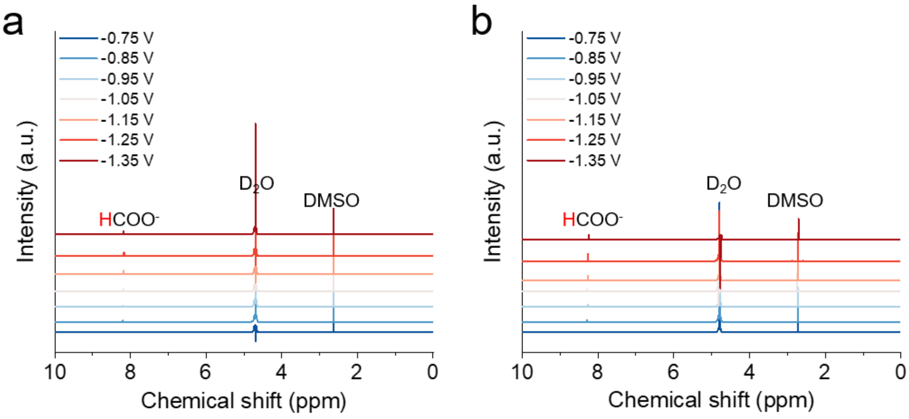

Figure S8. NMR spectra of the electrolyte obtained after one hour of electrolysis using (a) B-SnO_2 and (b) M-SnO_2.

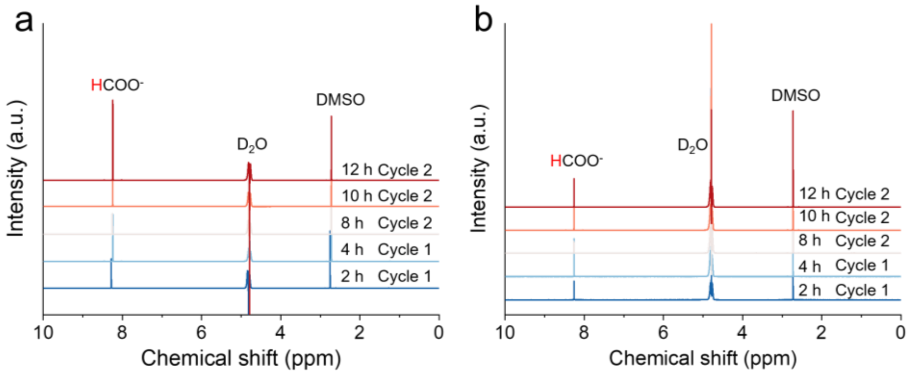

Figure S9. NMR spectra of the electrolyte collected after different durations of the CO_2RR stability test using (a) M-SnO_2 and (b) B-SnO_2 at a constant potential of -1.15 V.

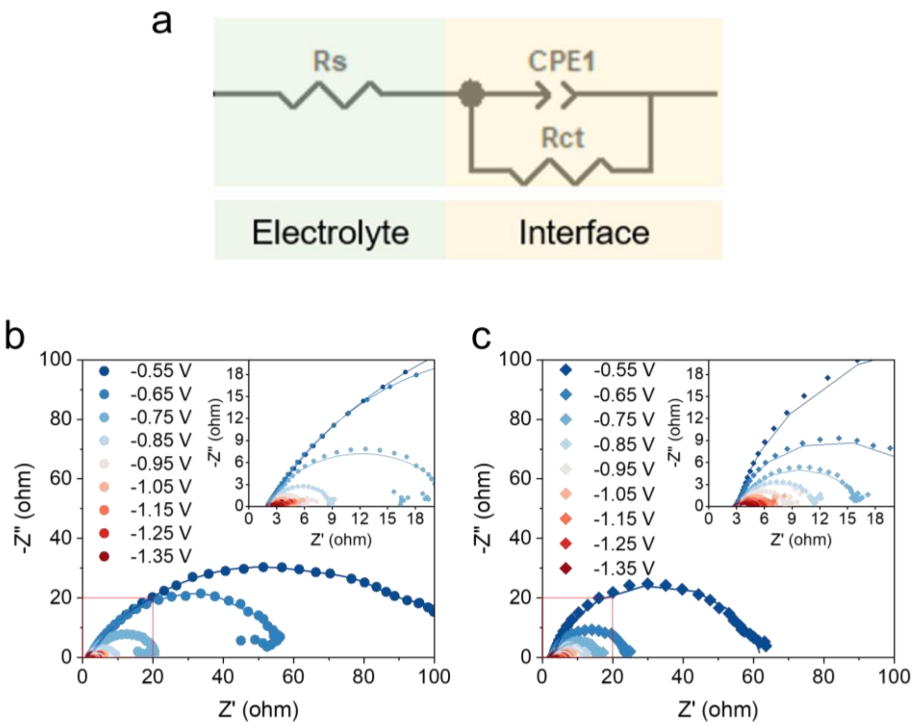

Figure S10. (a) The EIS fitting circuit includes three components: R_s, representing the solution resistance; CPE1, denoting the constant phase element; and R_{ct}, indicating the interfacial charge transfer resistance. EIS measurements at various applied potentials of (b) M-SnO_2 and (c) B-SnO_2 in CO_2-saturated 0.5 M $KHCO_3$, with solid lines representing fitted curves.

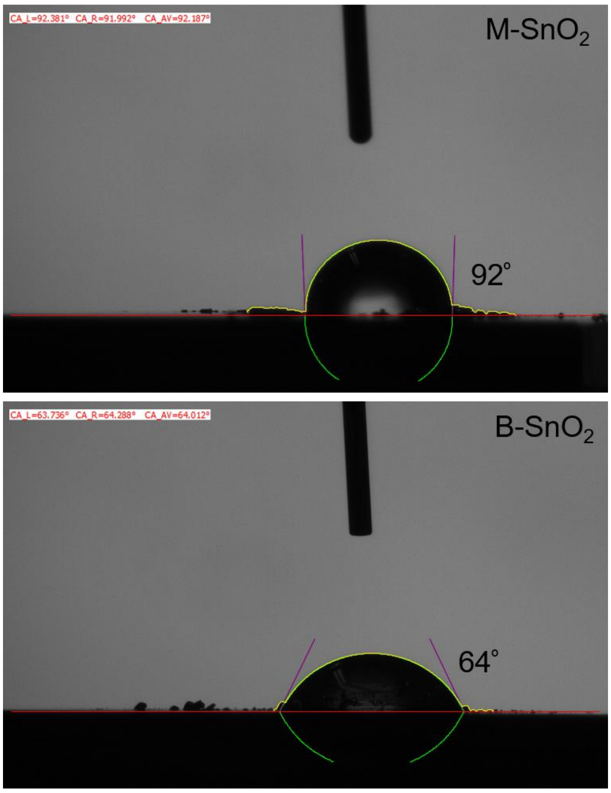

Figure S11. Contact angles of M-SnO_2 and B-SnO_2.

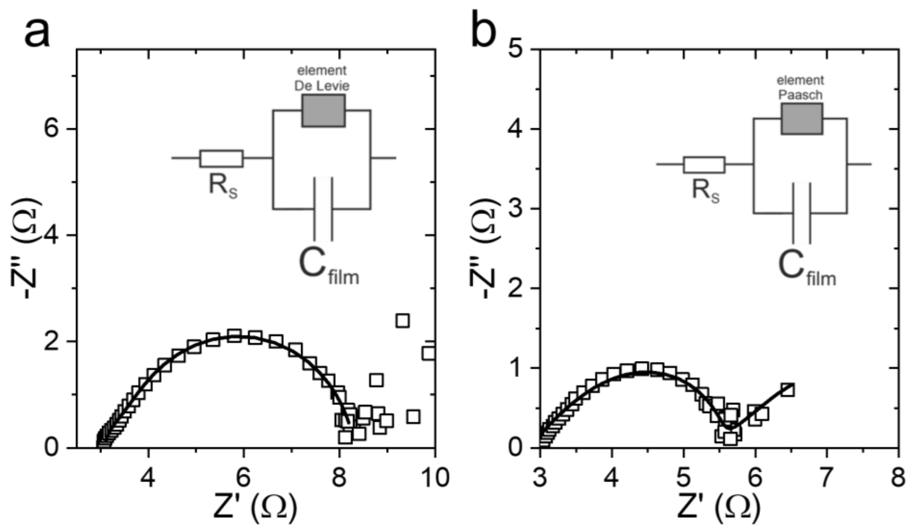

Figure S12. The impedance spectra of electrode modified by B-SnO_2 at -0.95 V (a) and -1.15 V (b) Insets: equivalent circuits utilized for fitting (as an example of how the fitting was performed).

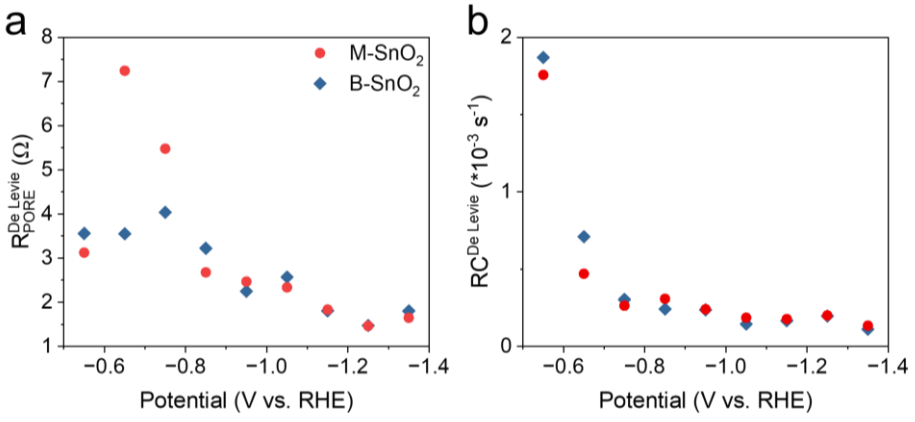

Figure S13. The potential dependences of pore resistance (a) and RC (b) estimated from the circuit with finite length De Levie element (inset of Figure S12a).

Supplementary Note 2: An equivalent circuit developed for reactive and conductive cylindrical pores, so called De Levie pores, was utilized.[9, 10] Here, the equivalent circuit consists of solution resistance in series with element containing finite De Levie element in parallel with the film capacitor (inset in Figure S12a). Good fit was obtained only at the frequencies above 10 Hz (Table S3). Due to the unknown number of pores, the kinetics of the faradaic process can be represented by RC constant instead of single fitted parameter.[11] The dependence of RC constant on the potential showed minor effect of mesoporosity except the onset potential of the general faradaic reactions. The use of the equivalent circuit with element proposed by Paasch[12] (inset of Figure S12b) enabled to fit a whole spectrum in a wide frequency region.

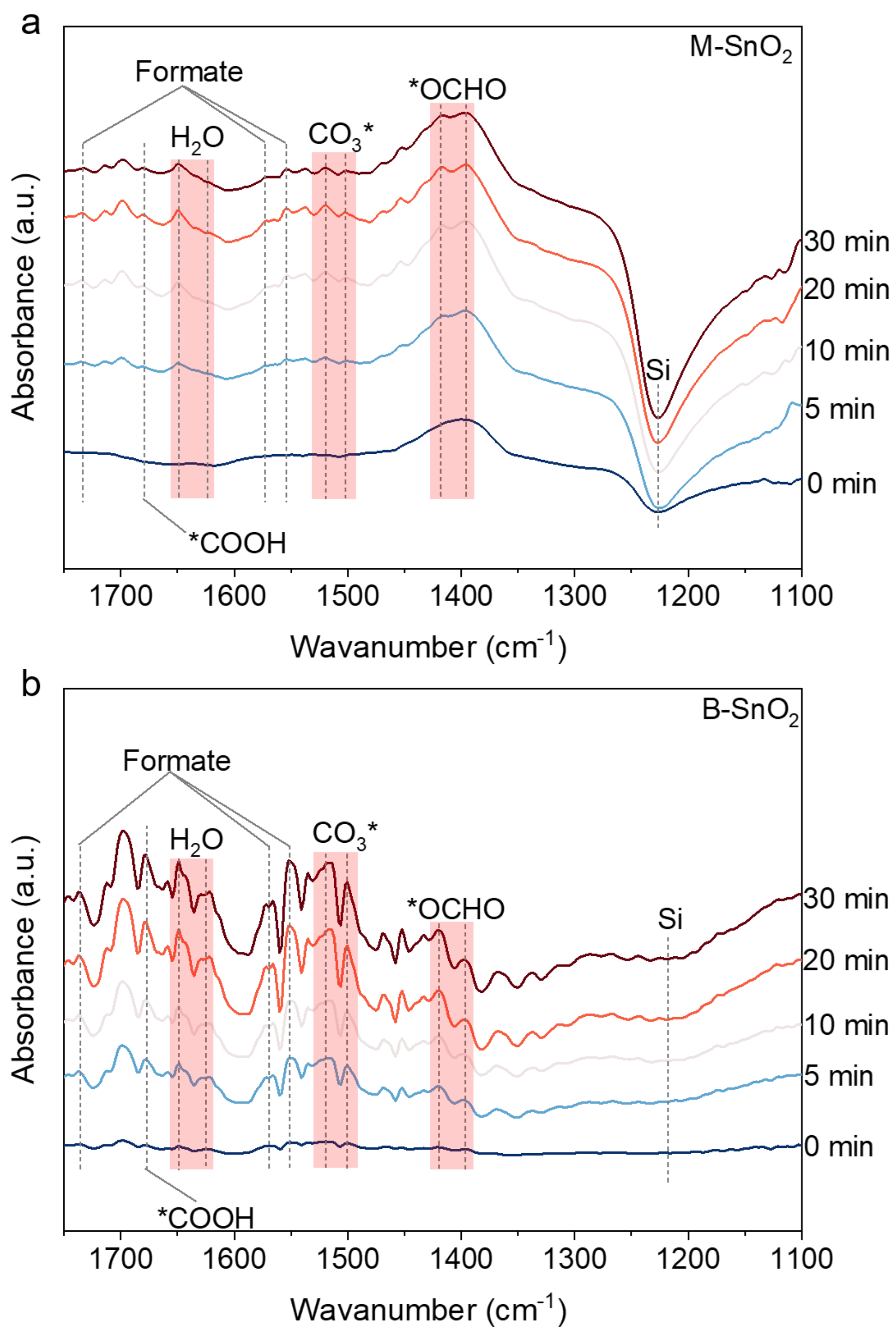

Figure S14. Time-dependent in situ ATR-SEIRAS of (a) M-SnO_2 and (b) B-SnO_2.

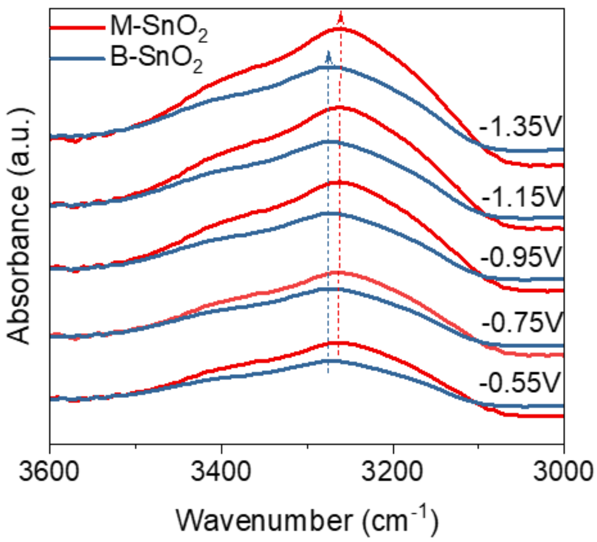

Figure S15. In situ ATR-SEIRAS of interfacial water over M-SnO_2 and B-SnO_2 under different applied potentials.

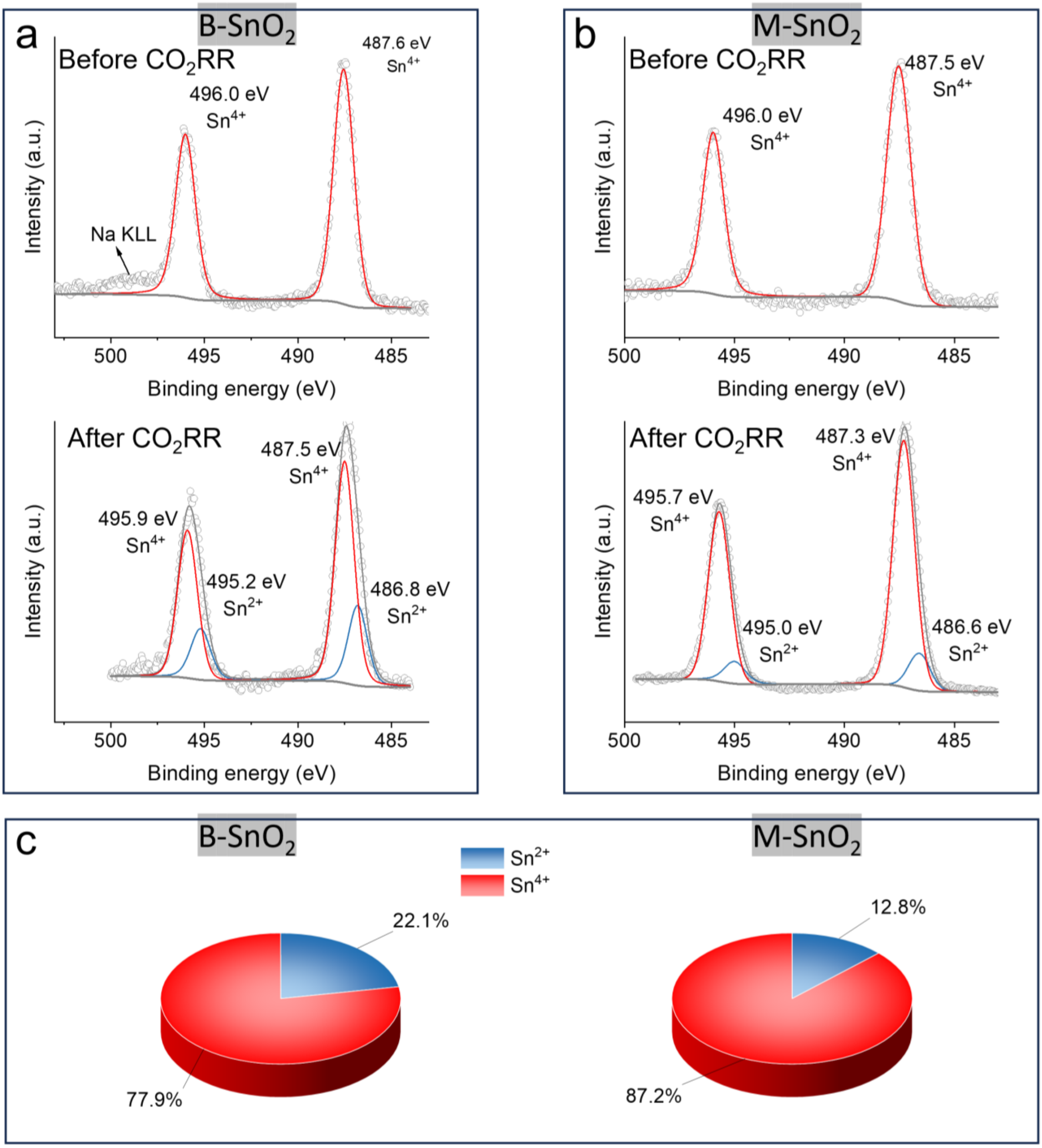

Figure S16. Sn 3d XPS spectra of (a) B-SnO_2 and (b) M-SnO_2 before and after CO_2RR. (c) The proportion of Sn^{2+}/Sn^{4+} of B-SnO_2 and M-SnO_2 after CO_2RR (1.15 V vs. RHE, 12 h**).**

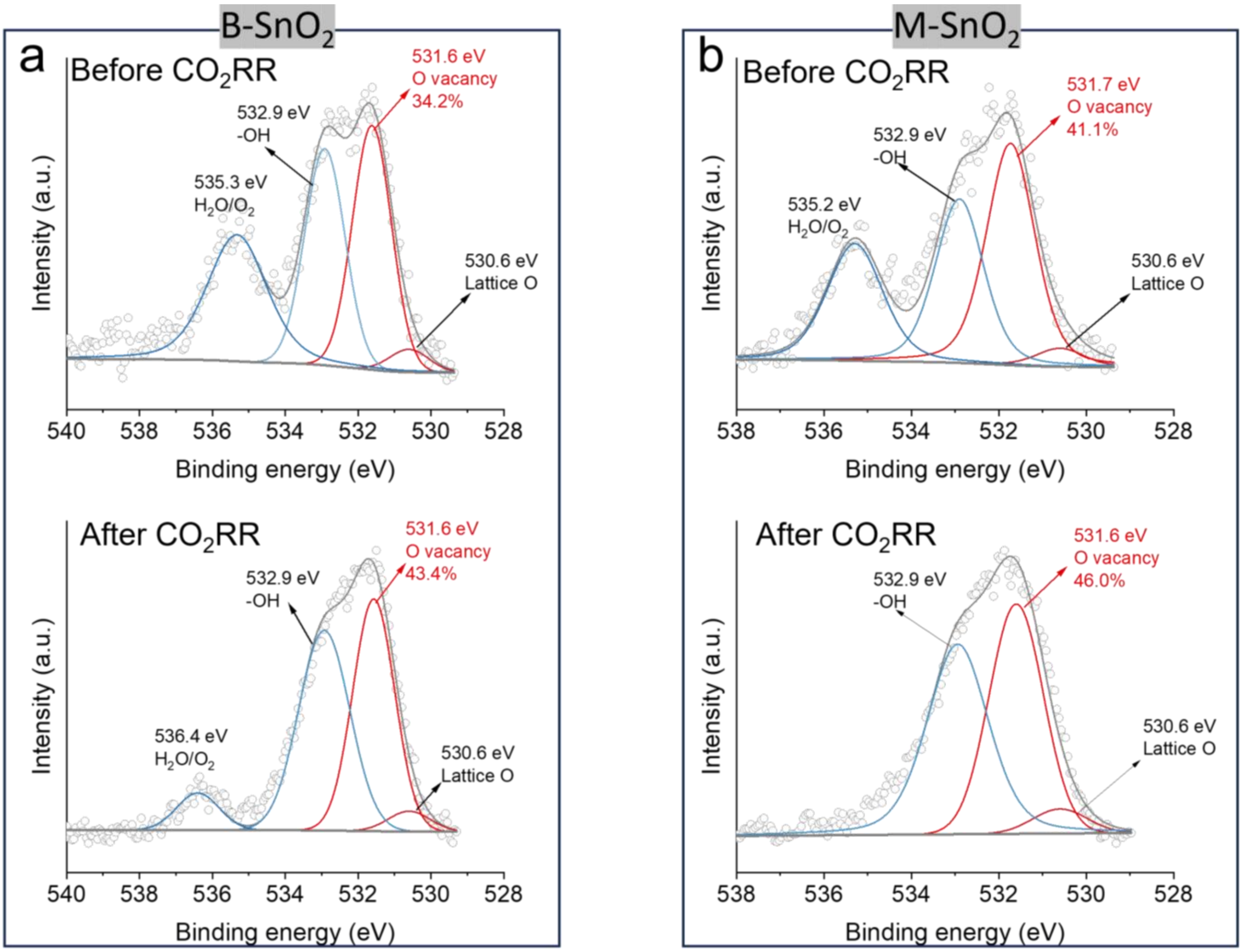

Figure S17. O 1s XPS spectra of (a) B-SnO_2 and (b) M-SnO_2 before and after CO_2RR.

Supplementary Note 3: To investigate the electronic structure changes before and after CO_2RR, XPS measurements were performed on the electrodes. As shown in Figure S17, the concentration of oxygen vacancies in M-SnO_2 is higher than that in B-SnO_2, which is consistent with the EPR results in Figure 2f. After CO_2RR, partial reduction of Sn^{4+} occurred along with structural reconstruction, leading to the formation of additional oxygen vacancies.

To further examine the Sn valence state, the Sn 3d XPS spectra were analyzed (Figure S16). The fitting results reveal that, after the reaction, M-SnO_2 retains a higher proportion of Sn^{4+} compared with B-SnO_2.[13, 14] This suggests that the presence of oxygen vacancies helps stabilize Sn against over-reduction during CO_2RR. The higher oxygen vacancy concentration observed in M-SnO_2 after CO_2RR further supports this conclusion.[8]

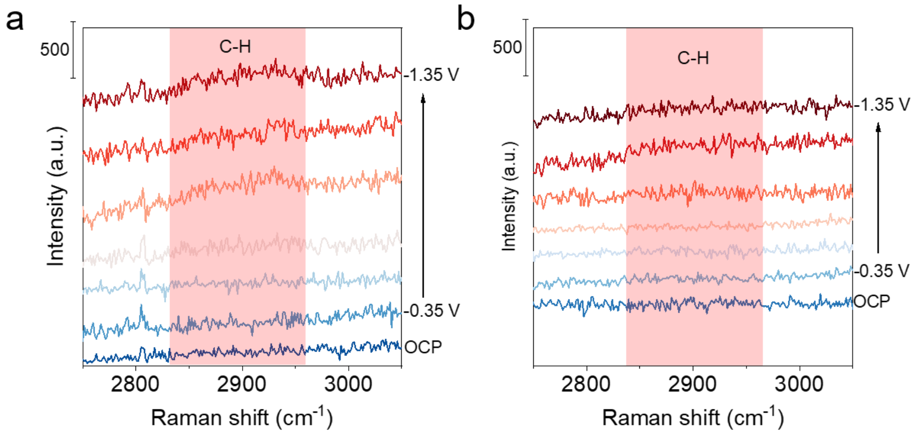

Figure S18. In situ SERS of C-H on the (a) M-SnO_2 and (b) B-SnO_2.

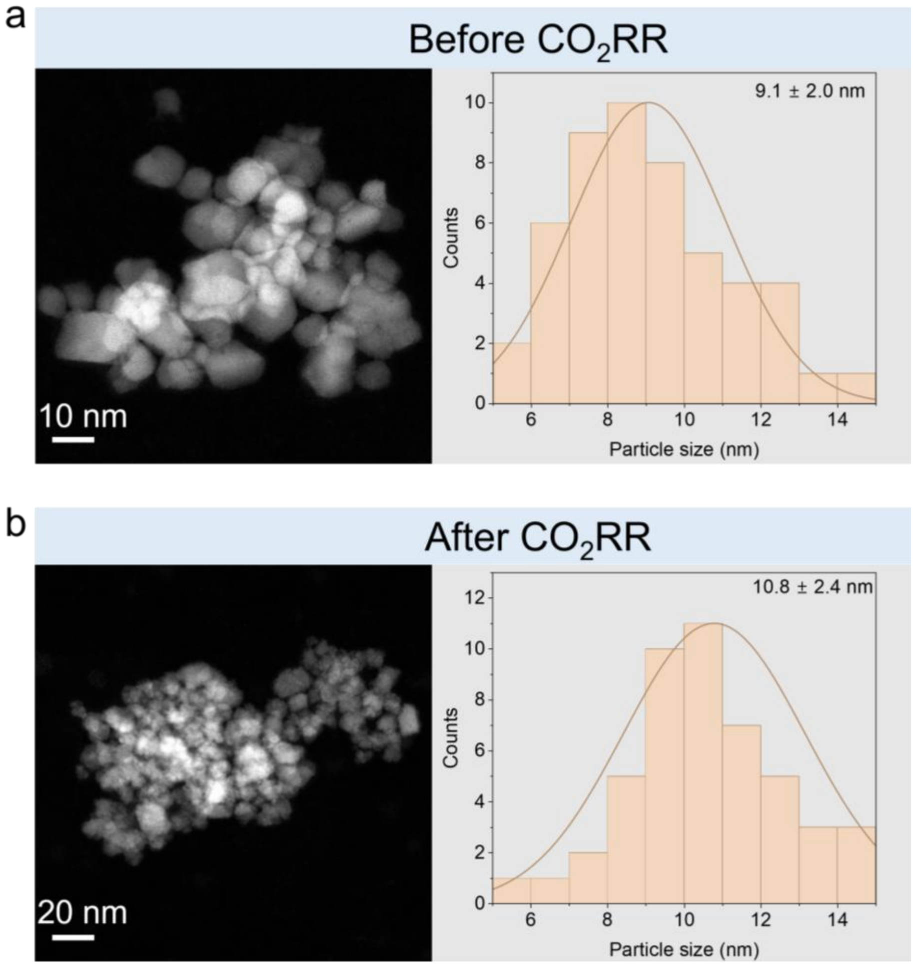

Figure S19. HAADF-STEM micrographs of M-SnO_2 before and after CO_2RR, accompanied by an analysis of particle size evolution.

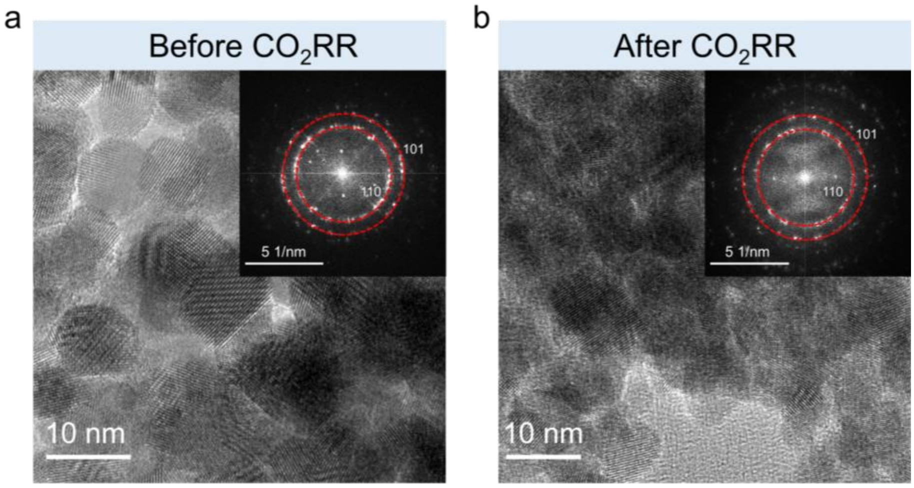

Figure S20. HR-TEM micrographs of M-SnO_2 (a) before and (b) after CO_2RR and the corresponding FFT patterns.

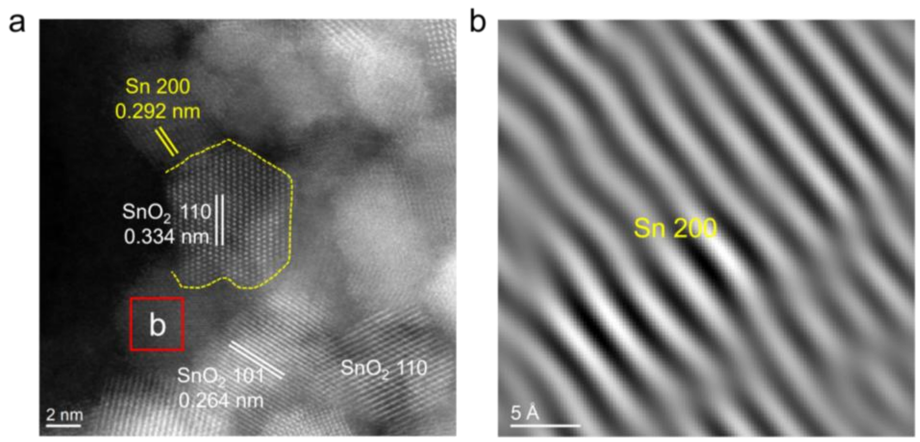

Figure S21. HR HAADF-STEM micrographs of (a) M-SnO_2 and (b) corresponding inverse fast Fourier transform (IFFT) patterns from (a).

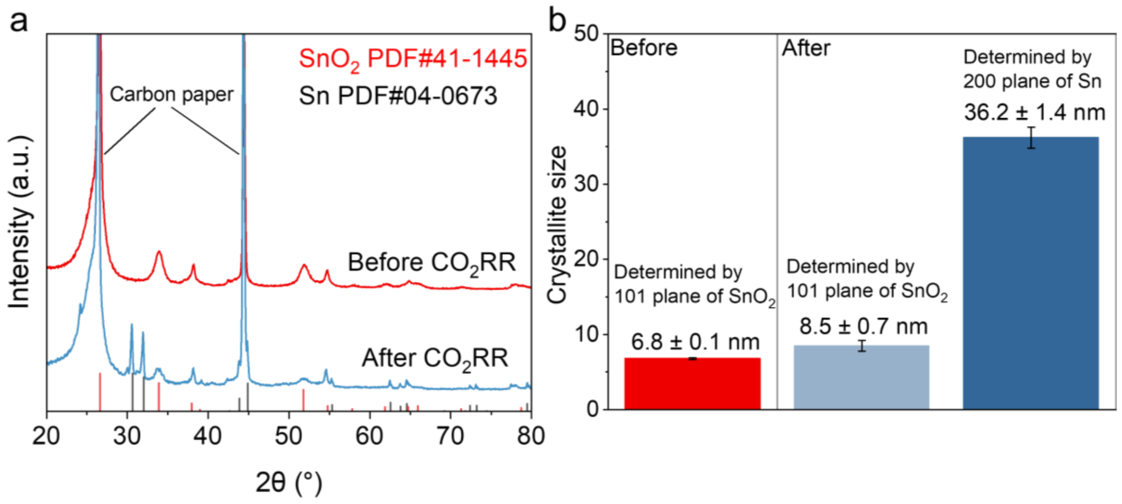

Figure S22. (a) X-ray diffractograms of M-SnO_2 before and after CO_2RR. (b) Comparation of crystallite size of M-SnO_2 before and after CO_2RR.

Supplementary Note 4: As shown in Figure S22, diffraction peaks corresponding to metallic Sn appear after CO_2RR, matching well with the standard Sn XRD pattern (PDF #04-0673). This result indicates that part of M-SnO_2 was reduced to metallic Sn, which is consistent with the STEM observations of M-SnO_2 after CO_2RR. To estimate the crystalline domain size, XRD peaks in the 2θ range of 30°–36° were fitted using Gaussian functions. The associated error bars were derived from the standard errors of the full width at half maximum (FWHM) obtained from the fitting. The coherent crystalline domain size was then calculated using the Scherrer equation as follows:

$$D = \frac{K\lambda}{\beta cos\theta}$$

Where K is dimensionless shape factor (0.9), λ is the X-ray wavelength, β is the FWHM of the fitted planes, and θ is the corresponding diffraction angle.

2. Supplementary Tables

Table S1. EDS mapping elements summary of M-SnO_2

Element	series	Net	[wt.%]	[norm. wt.%]	[norm. at. %]
Tin	L-series	6299	67.7	67.7	22.0
Oxygen	K-series	4788	32.3	32.3	78.0

Table S2. The parameters of impedance spectra fitting by R-CPE circuit.

Catalyst	Potential (V vs. RHE)	R_s	CPE-T	CPE-P	R_{ct}
M-SnO_2	-0.55	1.925	0.0012341	0.69481	102.1
	-0.65	1.984	0.00073103	0.7695	57.46
	-0.75	2.092	0.0010023	0.78531	20.31
	-0.85	2.185	0.0012782	0.82809	6.934
	-0.95	2.171	0.0012444	0.84339	3.862
	-1.05	2.137	0.0013838	0.83686	2.682
	-1.15	2.103	0.0016303	0.82417	1.942
	-1.25	2.091	0.0020204	0.80772	1.486
	-1.35	2.095	0.0016958	0.84066	1.149
B-SnO_2	-0.55	2.999	0.00073581	0.87481	59.08
	-0.65	2.998	0.00074942	0.87904	21.59
	-0.75	3.213	0.0011128	0.82098	13.29
	-0.85	3.096	0.0012569	0.80866	8.532
	-0.95	3.119	0.0012589	0.819	5.318
	-1.05	3.049	0.0014866	0.79939	3.703
	-1.15	3.03	0.0016359	0.79246	2.679
	-1.25	3.003	0.0019563	0.78545	2.144
	-1.35	2.978	0.001732	0.81487	1.594

Table S3. The parameters of impedance spectra fitting by circuit with De Levie element.

Catalyst	Potential (V vs. RHE)	R_S (Ω)	A	B (×10^{-3})	ϕ	R_{PORE} (Ω)	C (×10^{-3} mF)	RC[1] (×10^{-3} s^{-1})
B-SnO_2	-0.55	2.89	15.64	1.899	0.88	3.56	0.12	1.871
	-0.65	2.90	5.65	1.854	0.90	3.55	0.13	0.71
	-0.75	3.13	2.89	3.04	0.88	4.04	0.11	0.3
	-0.85	3.04	2.29	2.884	0.87	3.22	0.11	0.242
	-0.95	3.07	2.04	2.407	0.85	2.25	0.12	0.24
	-1.05	3.05	1.08	2.817	0.87	2.57	0.14	0.145
	-1.15	3.04	1.14	3.039	0.81	1.81	0.15	0.166
	-1.25	3.02	1.11	3.568	0.77	1.47	0.18	0.197
	-1.35	2.98	0.57	4.229	0.83	1.80	0.19	0.111
M-SnO_2	-0.55	2.09	33.77	4.506	0.64	3.12	0.052	1.757
	-0.65	2.05	7,64	4.91	0.75	7.24	0.061	0.47
	-0.75	2.07	3.20	3.646	0.85	5.48	0.082	0.264
	-0.85	2.14	2.21	2.42	0.88	2.68	0.139	0.308
	-0.95	2.16	1.22	2.648	0.86	2.47	0.197	0.24
	-1.05	2.14	0.82	3.641	0.83	2.34	0.227	0.186
	-1.15	2.12	0.72	4.214	0.79	1.83	0.245	0.177
	-1.25	2.12	0.69	8.151	0.68	1.46	0.293	0.2
	-1.35	2.11	0.42	10.017	0.71	1.65	0.319	0.134

[1]Calculated as: $RC = AC$

3. References

(1) Ma, T. Y.; Zheng, Y.; Dai, S.; Jaroniec, M.; Qiao, S. Z. Mesoporous $MnCo_2O_4$ with abundant oxygen vacancy defects as high-performance oxygen reduction catalysts. *J. Mater. Chem. A* **2014**, *2* , 8676.

(2) Wang, D.; Sun, J.; Cao, X.; Zhu, Y.; Wang, Q.; Wang, G.; Han, Y.; Lu, G.; Pang, G.; Feng, S. High-performance gas sensing achieved by mesoporous tungsten oxide mesocrystals with increased oxygen vacancies. *J. Mater. Chem. A* **2013**, *1*, 8653.

(3) Jaiswal, A.; karri, s. s. v. p. r.; Kang, S. G.; Kumar, R.; Hur, S. H. Tailoring Defect Generation in SnO_2 Nanostructure for Increased Selectivity in Electrochemical CO_2 Reduction. *Nanoscale* **2025**, 17. 22467.

(4) Liu, G.; Li, Z.; Shi, J.; Sun, K.; Ji, Y.; Wang, Z.; Qiu, Y.; Liu, Y.; Wang, Z.; Hu, P. Black reduced porous SnO_2 nanosheets for CO2 electroreduction with high formate selectivity and low overpotential. *Appl. Catal. B: Environ.* **2020**, *260*, 118134.

(5) Ye, F.; Zhang, S.; Cheng, Q.; Long, Y.; Liu, D.; Paul, R.; Fang, Y.; Su, Y.; Qu, L.; Dai, L.; et al. The role of oxygen-vacancy in bifunctional indium oxyhydroxide catalysts for electrochemical coupling of biomass valorization with CO_2 conversion. *Nat. Commun.* **2023**, *14*, 2040.

(6) Dong, W.; Xu, J.; Wang, C.; Lu, Y.; Liu, X.; Wang, X.; Yuan, X.; Wang, Z.; Lin, T.; Sui, M.; et al. A Robust and Conductive Black Tin Oxide Nanostructure Makes Efficient Lithium-Ion Batteries Possible. *Adv. Mater.* **2017**, *29*, 1700136.

(7) Wang, G.; Xiao, X.; Li, W.; Lin, Z.; Zhao, Z.; Chen, C.; Wang, C.; Li, Y.; Huang, X.; Miao, L.; et al. Significantly Enhanced Visible Light Photoelectrochemical Activity in TiO_2 Nanowire Arrays by Nitrogen Implantation. *Nano Lett.* **2015**, *15*, 4692.

(8) Jiang, Y.; Shan, J.; Wang, P.; Huang, L.; Zheng, Y.; Qiao, S.-Z. Stabilizing Oxidation State of SnO2 for Highly Selective CO2 Electroreduction to Formate at Large Current Densities. *ACS Catal.* **2023**, *13*, 3101.

(9) de Levie, R. On porous electrodes in electrolyte solutions: I. Capacitance effects. *Electrochim. Acta* **1963**, *8*, 751.

(10) De Levie, R. The influence of surface roughness of solid electrodes on electrochemical measurements. *Electrochim. Acta* **1965**, *10*, 113.

(11) Lasia, A. Impedance of porous electrodes. *J. Electroanal. Chem.* **1995**, *397*, 27.

(12) Paasch, G.; Micka, K.; Gersdorf, P. Theory of the electrochemical impedance of macrohomogeneous porous electrodes. *Electrochim. Acta* **1993**, *38*, 2653.

(13) Liu, H.; Li, B.; Liu, Z.; Liang, Z.; Chuai, H.; Wang, H.; Lou, S. N.; Su, Y.; Zhang, S.; Ma, X. Ceria -Mediated Dynamic $Sn^0/Sn^{\delta+}$ Redox Cycle for CO_2 Electroreduction. *ACS Catal.* **2023**, *13*, 5033.

(14) Chen, Y.; Kanan, M. W. Tin Oxide Dependence of the CO_2 Reduction Efficiency on Tin Electrodes and Enhanced Activity for Tin/Tin Oxide Thin-Film Catalysts. *J. Am. Chem. Soc.* **2012**, *134*, 1986.
